# When a child lives with atopic dermatitis: an integrative literature review on parental experience

**DOI:** 10.3389/fped.2025.1720595

**Published:** 2025-12-10

**Authors:** Chiara Martis, Annalisa Levante, Flavia Lecciso

**Affiliations:** 1 Department of Human and Social Sciences, University of Salento, Lecce, Italy; 2 Laboratory of Applied Psychology, Department of Human and Social Sciences, University of Salento, Lecce, Italy

**Keywords:** atopic dermatitis, eczema, chronic disease, parent-child relationship, parents as workers, parents as individuals, parents as caregivers

## Abstract

This integrative literature review, conducted with a systematic approach, examined how a child’s atopic dermatitis affects parental functioning. Fifty-four studies have been reviewed, identifying the key impacts, including work absenteeism, reduced quality of life, increased distress, sleep deprivation, and challenges in parent-child relationships. These dimensions were grouped into three focuses: Parents as workers, individuals, and caregivers. The review found that atopic dermatitis often disrupts parents' careers, causes emotional strain, family burden, and sleep issues. Parent-child interactions may also be affected, potentially impacting the attachment bond. Overall, the findings highlighted the interconnected nature of parental experiences and stressed the need to consider all family members' perspectives. A systemic approach in clinical practice, policy, and research is crucial to better support parents managing their child's condition.

## 1 Introduction

Atopic Dermatitis (henceforth AD), or atopic eczema, is a chronic inflammatory skin disease characterised by scaly lesions, intense itching, and skin rash (1). It is a non-contagious, relapsing condition that primarily affects individuals in early infancy (2), with approximately 70% of cases developing within the first year of life (3, 4). Globally, according to the National Health Interview Survey (5), the prevalence of AD in children and adolescents is 10.8%, making it one of the most common chronic pediatric diseases.

The impact of the disease on the patient's quality of life was extensively demonstrated: Literature reviews, as well as empirical studies, reported social embarrassment and isolation due to the persistent itching and/or visible skin lesions (6–8). In addition, depression, suicidal ideation (9–11), sleep disturbances (12), and challenges in daily life activities (13) have been reported as patients' outcomes. According to a comparative study (14), AD was reported as one of the chronic conditions associated with notable reductions in quality of life. It is worth noting that the caregiving burden in managing patients with AD significantly affects their parents' quality of life (15). Due to the continuous care (e.g., medications) and vigilance (e.g., monitoring the medication, avoiding environmental triggers, devoting attention to diet), parents have to restructure family routines, may need to take time off from work, and could suffer from sleep deprivation and/or disturbances. As a sequel, emotional and physical fatigue, financial strain, distress in terms of depressive symptoms, and anxiety increase (16–20). As primary caregivers of children with AD, mothers have often been the focus of research and appear to be particularly vulnerable to stress (16). In extreme cases, the AD-related caregiving burden may be associated with suicidal ideation among mothers compared to those of typically developing (henceforth TD) children (16).

Overall, this stressful frame due to chronic disease management results in poor adherence to the disease treatment. In this vein, families could benefit from clinical intervention programs promoting, for instance, effective coping strategies for managing the disease (16). In addition, due to the high cost of medications, financial support from public health services could be beneficial for families (8, 21).

Hence, an overview of what parental dimensions were affected by the child's AD is required. To our knowledge, no reviews have yet summarised the existing literature on the topic. To address this gap, the present integrative literature review aimed to identify the dimensions examined in parents of children with AD, leading to an appropriate categorisation from a lifespan perspective, and synthesise the main results narratively. In line with Jensen's (22) assumption, integrative review allows researchers not only to identify and critique the existing evidence systematically but also to integrate it in a way that generates new perspectives and conceptual frameworks. A hybrid approach was applied: firstly, to ensure methodological rigour, a systematic search was conducted (23) to identify all available empirical evidence aligned with the research topic (i.e., the impact of having a child with AD on parental functioning); secondly, a critical lens was adopted to synthesise the extracted literature and identify future directions. Investigating the topic could support the design of not only further research but also targeted interventions in this vulnerable population.

## 2 Methods

### 2.1 Search strategy

The current integrative literature review was conducted using a 4-stage structured approach consisting of (1) detecting the topic on which the integrative review focuses; (2) the literature search using a clear and replicable strategy to identify relevant studies to include in the review; (3) data analysis that organise, code, categorise, and synthesise the relevant information from the included papers into a coherent interpretation of the existing research; finally, (4) the presentation stage consists of reporting the findings, following the overarching directions developed during the analytical process. The systematic approach to the electronic search stage was adopted to ensure the retrieval of as many papers on the topic as possible.

All relevant documents published up to January 23rd, 2025, were included. The Preferred Reporting Items for Systematic Reviews and Meta-Analyses (PRISMA) framework (24) guided the search strategy and reporting. Seven databases were systematically searched: Scopus, MEDLINE, PsycINFO, CINAHL (Cumulative Index to Nursing and Allied Health Literature), ERIC (Education Resources Information Center), PubMed, and Web of Science. The following Boolean operators and keyword combinations were applied in each database: (atopic dermatitis OR eczema) AND (parent* OR caregiver* OR mother* OR father*) AND (impact OR experience).

The studies were filtered by subject areas of psychology, medicine, nursing, multidisciplinary research, and social sciences; no filter by the country where the study was conducted was applied. The inclusion criteria were as follows: (a) papers published up to January 2025; (b) papers published in peer-reviewed and indexed journals; (c) papers written in English or Italian; (d) papers focusing on the impact of a child's atopic dermatitis on parents; (e) quantitative, qualitative, and mixed-method studies. The exclusion criteria were: (a) papers involving only parents of adult children (>18 years old); (b) papers focused on non-psychological dimensions (i.e., medical issues); (c) systematic, scoping, integrative, and narrative reviews; (d) validation studies on measures assessing the impact of the AD in parents; (e) papers examining the effectiveness of intervention programs; (f) papers focusing on the impact of disabilities and/or chronic disease on other family members (e.g., siblings).

### 2.2 Selection of the studies

After identifying the central topic addressed by the review and defining the search strategy, the literature search stage was conducted in accordance with the PRISMA checklist (24).

**Figure 1:**
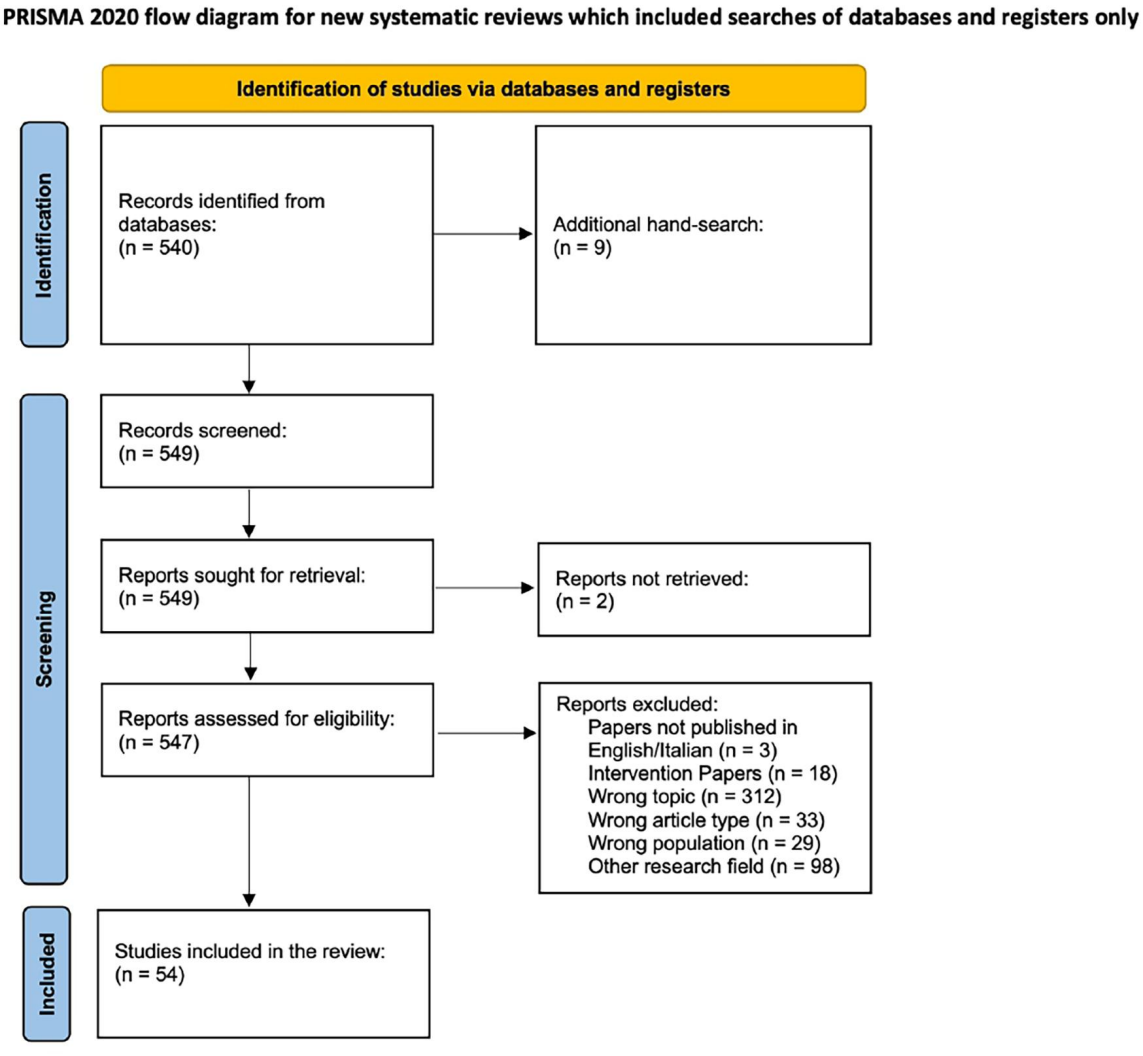
PRISMA flow diagram.

Figure 1 maps the papers' selection process. Following the Identification stage of PRISMA, the papers in which the pre-selected keywords appeared in either the title, abstract, subject heading, or keywords list have been searched. All database outputs (540 records) were tabulated in the Excel spreadsheet alphabetically. An additional hand search was performed to incorporate a further 9 records according to the reference lists of reviewed studies. Following the Screening stage of the PRISMA, a total of 549 papers were screened by two authors by reading titles and abstracts (CM & AL). Two papers were excluded because the full text could not be retrieved. According to the pre-defined inclusion and exclusion criteria, 493 papers have been removed. In case of disagreement, the third author (FL) arbitrated. The inter-rater agreement was excellent (Cohen's *k* = 0.90).

Finally, 54 papers (marked with * in the reference list) were included in the integrative literature review.

## 3 Results

The section allows for reporting the main results of data analysis and presentation stages. The data analysis stage was guided by the theoretical framework that supported the identification of overarching focuses and the organisation of recurring dimensions across studies. In the presentation stage, the findings are reported both descriptively, through a summary table and a figure outlining the methodological characteristics and key results, and narratively, following the analytical directions established during the data analysis stage.

### 3.1 Preliminary and descriptive study information

Given the large number of studies included, a two-step and top-down analytical strategy was adopted to analyse the data and ensure a systematic and conceptually coherent organisation of the evidence. The use of a top-down approach ensured that the emerging focuses reflected both the empirical evidence and its conceptual underpinnings. The approach made it possible to move from a broad body of literature to a structured set of thematic areas. In Step 1, each paper was read deeply to extract the specific dimensions examined in relation to the functioning of parents of children with AD. This step aimed to identify not only the primary dimensions investigated but also any additional dimensions that contributed to understanding parental functioning. In Step 2, the extracted dimensions were categorised into theoretically meaningful focuses. This categorisation was guided by a conceptual framework that enabled the clustering of diverse findings into higher-order areas of parental functioning. Once each dimension was categorised, a narrative synthesis was conducted to integrate the evidence across studies. This synthesis facilitated the identification of recurring patterns and the development of a multifaceted understanding of parental functioning in the context of childhood AD.

To be accurate, Step 1 allowed for the extraction of the following dimensions: Work absenteeism, quality of life, family burden (in terms of caregiving and management demands, role restrictions, social isolation), stress, anxiety, depression, hopelessness, coping strategies, parental experience, sleep quality, and the parent-child relationship. Several studies investigated mainly one dimension, while other ones tested the association/relation between more than one dimension. When further dimensions have been explored in the study, they have been reported as additional dimensions.

Following Step 2, each dimension was categorised into a corresponding focus designed under Super's life-career rainbow and adaptability model (25). The author stated that individuals assume multiple roles across life contexts (e.g., family, school, social, and professional) and throughout life stages (e.g., childhood, adolescence, adulthood). These roles are not biologically determined but are shaped by social and psychological factors, which interact and influence one another. For instance, adults often simultaneously take on the roles of children, parents, siblings, workers, and friends. This perspective is particularly relevant when considering the experiences of parents of children with AD, as their caregiving responsibilities are nested with other roles in their daily lives. Guided by this framework, three key focuses of parental functioning in the context of AD were identified: (1) Parents as workers, (2) parents as individuals, and (3) parents as caregivers. The first focus highlights how a child's AD impacts the caregiver's role as an adult worker. The second focus emphasises the various psychological dimensions of an adult that are influenced by their child's AD. Lastly, the third focus underscores how a child's AD directly affects the quality of the parent-child relationship.

Supplementary Table S1 (see Supplementary Materials) provides an overview of the descriptive information extracted from each reviewed study, organised according to the three overarching focuses. For each study, Supplementary Table S1 reports the main dimension examined (and any additional dimensions, where applicable), the reference [author(s) and year of publication], and the country in which the research was conducted, classified as Global North (i.e., developed countries) or Global South (i.e., developing countries). Methodological details are also included, such as the study design (cross-sectional or longitudinal), the research methodology (quantitative, qualitative, or mixed), and participant characteristics (sample size, sex distribution, mean age with standard deviation, and parental and child age ranges).

Lastly, Supplementary Table S1 lists the measures administered in each study, accompanied by a brief description of what each instrument assesses. The study aims and main findings are summarised to offer a picture of the evidence reviewed.

Following this structure, the results of the integrative literature review are presented below. First, the methodological characteristics of the included studies have been outlined; then, the main results are discussed concerning the three focuses of parental functioning.

### 3.2 Methodological characteristics of the reviewed studies

This paragraph summarises the methodological characteristics of the reviewed studies according to the focus. The synthesis was reported according to the information reported in Supplementary Table S1.

A total of 54 studies were included: 1 study related to the parents as workers, 48 studies related to the parents as individuals, and 5 studies related to the parents as caregivers.

Regarding the first focus of functioning, i.e., parents as workers, only one study conducted by Cheng et al. (26) was included. This cross-sectional quantitative study was carried out in the Global North. A total of 124,267 participants were included. The study compared parents of children with AD, parents of children with psoriasis, and parents of TD children. No information was provided on the sex of parents and children, nor the age range of parents. The children's ages ranged from 3 to 22 years. The study used an *ad hoc* questionnaire for data collection.

Regarding the second focus of functioning, i.e., parents as individuals, 48 studies were included. The majority (*n* = 28) were conducted in Global North countries, while 16 involved participants from Global South countries, and four included data from both regions. Concerning study design, 45 studies were cross-sectional, while three were longitudinal. Regarding methodology, 40 studies were quantitative, seven were qualitative, and one study used a mixed-methods approach. Sample sizes varied considerably across studies, with quantitative studies ranging from 49 to 10,099 participants and qualitative studies ranging from 9 to 77 participants. The sample size for the mixed-method study was 32 parents. A total of 11 studies (16, 17, 27–35) compared a clinical group (i.e., parents of children with AD) to a control group (parents of TD children). The study by Moore et al. (18) compared parents of children with AD to those of children with asthma. Finally, Meltzer & Booster (36) examined parents of children with AD, asthma, and ventilator-assisted children, as well as parents of TD children. 13 studies involved only mothers, while 18 studies included both parents, with a strong predominance of mothers. The remaining 17 studies did not report the parental sex distribution. The ages of parents in the reviewed studies ranged from 15 to 64 years (although in most studies, the age range was not reported). Regarding the child's sex, although some studies did not report sex distribution, the majority included a balanced sample, while the ages of the children/adolescents ranged from 0 to 20 years. Standardised questionnaires were the most commonly used data collection method in quantitative studies, while semi-structured interviews were predominantly employed in qualitative research. The mixed-method study utilised both standardised questionnaires and *ad hoc* interviews.

Lastly, regarding the third focus of functioning, i.e., parents as caregivers, 5 studies were included. Four of them were conducted in Global North countries, while one study involved participants from the Global South. Concerning study design, four studies were cross-sectional, while 1 adopted a longitudinal design. Regarding methodology, 4 studies were quantitative, and 1 used a mixed-methods approach. Sample sizes ranged from 40 to 242 participants, with the sample size for the mixed-method study being 97 parents.

Two studies (37, 38) compared a clinical group, consisting of parents of children with AD, to a control group of parents of TD children, while the study by Cassibba et al. (39) compared different clinical groups (children with AD and premature ones) with a control group.

In terms of parental participation, 4 studies involved only mothers, and only 1 study included both mothers and fathers, with a strong predominance of mothers. Concerning the children, the studies generally included a fairly balanced sample, except one study, which showed a higher prevalence of males. Not all studies reported the details of the parents' and children's age ranges. Among those who provided this information, the parental age ranged from 19 to 42 years, while the age of the children/adolescents ranged from 5 months to 12 years. As in the previous focuses, standardised questionnaires were the most commonly used method for data collection.

### 3.3 Focuses

The main results of the studies are discussed in the following sections. As previously highlighted, to narratively and critically synthesise the reviewed studies, the extracted dimensions have been categorised into three main focuses, each representing a specific area of parental functioning affected by having a child with AD: Work, individual, and caregiving functioning. It is worth noting that the dimensions are deeply interconnected, making it challenging to categorise them separately. For instance, the demands of caring for a child with AD require significant parental involvement, which may lead to reduced quality of life, increased stress, anxiety, sleep problems, as well as difficulties in the workplace or the parent-child relationship. Fatigue and overwhelming problems at work may increase stress, and vice versa.

Therefore, while the results will be discussed within the three predefined focuses, in line with Super's theoretical model (25), it is important to acknowledge that each focus is closely interconnected with the others.

#### 3.3.1 Parents as workers

Only the study by Cheng et al. (26) focused mainly on parental challenges in work life due to the management of children with AD. The study examined parental work absenteeism, and it found a positive association between the chronic absenteeism of children with AD from school and the corresponding work absenteeism in their parents. Parents of children with AD were more likely to miss work, with higher rates of absenteeism reported in comparison to parents of children with psoriasis and TD children. In addition, the study revealed that parents missed more days of work annually, primarily due to their caregiving responsibilities. Cheng et al. (26) concluded that the caregiving demands of children with AD not only disrupted the children's school and social lives but also had a considerable economic impact on parents, as they missed work and experienced professional consequences due to their caregiving role.

As reported above, the dimensions are nested within them. Indeed, studies explored the parental quality of life as well as mental health, and also investigated the impact of the child's disease on parental work. For instance, the study by Yamaguchi et al. (40) pointed out that full-time working mothers demonstrated a heightened vulnerability to parental stress, likely due to the challenges of balancing work commitments and family responsibilities. Marciniak et al. (20) revealed a strong interference of the child's disease in paternal work life: Fathers reported significant disruptions to their professional activities, which could be attributed to the increased caregiving demands associated with their child's condition. Neri et al. (32) highlighted that parents often feel overwhelmed by the daily caregiving demands, with some even reporting changes to their work life. Specifically, 1 in 10 parents in their study had to alter their career paths or change jobs to manage the challenges posed by caring for a child with AD. In addition, the study by Cheung and Lee (41) reported the sacrifices parents make when caring for a child with AD: Some parents were forced to leave their jobs, while others reported sacrificing personal goals, such as pursuing pregnancy, engaging in leisure activities, or maintaining their social life.

#### 3.3.2 Parents as individuals

Most of the reviewed studies fall within this focus. As they primarily address individual functioning, these studies explore closely related and, in some cases, overlapping dimensions, such as quality of life, family burden, stress, anxiety, depression, hopelessness, coping strategies, parental experiences, and sleep quality.

This focus encompasses all dimensions experienced by parents, both emotional (e.g., quality of life, stress, anxiety) and physical (e.g., sleep quality), and financial. Seven studies (41–47) explored the overall burden associated with raising a child with AD, including caregiving demands, role restrictions, social isolation, and conflicts with extended family (defined as relatives beyond the immediate household, such as grandparents, uncles, aunts, and cousins). Zarit et al. (48) conceptualized caregiving burden as “the extent to which caregivers perceived their emotional, physical health, social life, and financial status as a result of caring for their relative”. In the AD context, parents reported difficulties in multiple areas of life, including sleep disturbances, physical and emotional exhaustion, social isolation, and strained family relationships (41–47). They also expressed feelings of guilt, frustration, and self-criticism in their parental role, as well as fear and despair (41, 43, 46, 47). Their condition often interfered with daily activities and increased concerns about the future. Additionally, parents reported anxiety regarding appropriate treatment choices. Some also highlighted that medical professionals occasionally underestimated the severity of the disease and provided insufficient support (47), while others mentioned inadequate support from family and friends (41).

More specifically, most studies focused on parental quality of life, which is a multidimensional construct emphasising the individual's self-perceived well-being (49). It represents a subjective experience rather than an objective entity (50). In the context of AD, parental quality of life was compromised by frustration and concerns about the future (47), reduced social interactions (51), fear of long-term disease-related consequences for the child (52), heightened worry (52), financial strain (35, 52), increased childcare responsibilities and household duties (52, 53), and limited family leisure activities (41).

Quality of life is closely linked to caregivers' mental health, encompassing stress, anxiety, and depression. Mental health refers to a state of well-being in which individuals realise their potential, cope with life's stressors, and contribute productively to society (54). According to Keyes' model (55), mental health consists of three components: Emotional well-being (i.e., happiness, interest in life, satisfaction), psychological well-being (i.e., competence in managing daily responsibilities, positive social relationships, life satisfaction), and social well-being (i.e., social contribution, integration, actualisation, and coherence). Thus, mental health is not merely the absence of mental illness but a dynamic state of internal equilibrium enabling individuals to function harmoniously (56).

Unsurprisingly, mental health has been extensively studied among parents of children with AD: Studies revealed the high vulnerability of this population to stress (16, 28, 40, 57), anxiety (17, 18), and depression (17, 18).

Furthermore, one study (30) explored the role of coping strategies in parental stress management. Coping refers to the cognitive and behavioural efforts individuals employ to manage, tolerate, or mitigate stress and emotional distress in challenging situations (58) Despite the high parental levels of stress, parents often adopt positive coping strategies such as cognitive reinterpretation, emotional expression, and problem-focused coping, while some also resort to maladaptive strategies, including alcohol or drug use (30).

Another key area of investigation in this population is the quality of sleep. Research has demonstrated significant sleep deprivation and disruption among parents of children with AD. Harbottle et al. (59) found that mothers of children with moderate to severe AD experienced greater sleep difficulties, primarily due to increased childhood insomnia. These difficulties were further exacerbated by maternal concerns about the child's health, leading to both emotional and physical stress. Similarly, Ramirez et al. (34) reported that mothers of children with active AD (i.e., when the condition is currently flaring up or symptomatic) faced persistent challenges such as difficulty falling asleep, insufficient rest, and daytime exhaustion. Furthermore, Forer et al. (27) observed that parents of children with mild AD had shorter sleep durations compared to those of children with moderate to severe AD and a TD control group. Meltzer and Booster (36) highlighted that caregivers of children with chronic diseases, including AD, experienced significantly poorer sleep quality than those of children without chronic disease, with higher rates of insomnia and chronic sleep deprivation. These sleep disturbances appear to be driven not only by the necessity of responding to the child's needs but also by the overall stress associated with managing the illness.

Taken together, these challenges often lead parents (particularly mothers) to sacrifice personal interests and disengage from recreational and leisure activities, which could otherwise serve as protective factors in fostering psychological well-being and promoting a flourishing state of mind (55).

#### 3.3.3 Parents as caregivers

The child-parent relationship reflects the affective bond developed between parents (or caregivers) and the child. The high quality of this relationship has a long-term consequence for shaping the child's psychological functioning (60). It is a bidirectional relationship (60): On the one hand, parental sensitivity and responsiveness (61) impact the development of the child's internal representations of the self, the other, and the relationship; on the other hand, as children grow, their characteristics (e.g., temperament, external factors, …) may affect this relationship. Among the child-related factors that can impact the parent-child relationship, evidence highlighted the role of a child's chronic disease (62, 63). Some studies suggested that in the context of AD, parent-child interactions may present specific challenges, such as reduced eye contact and vocal communication (38).

Cassibba et al. (39) found that despite a higher prevalence of insecure attachment bonds among children with AD, maternal attachment representations and sensitivity did not significantly differ from those observed in the TD control group. Moreover, in clinical dyads, emotional availability appeared to be more strongly associated with maternal attachment representations than with the child's attachment security. Nevertheless, a study (37) revealed that a subgroup of mothers of children with AD perceived the additional caregiving demands as an opportunity to strengthen their bond with their child, fostering physical closeness and emotional intimacy.

**Figure 2:**
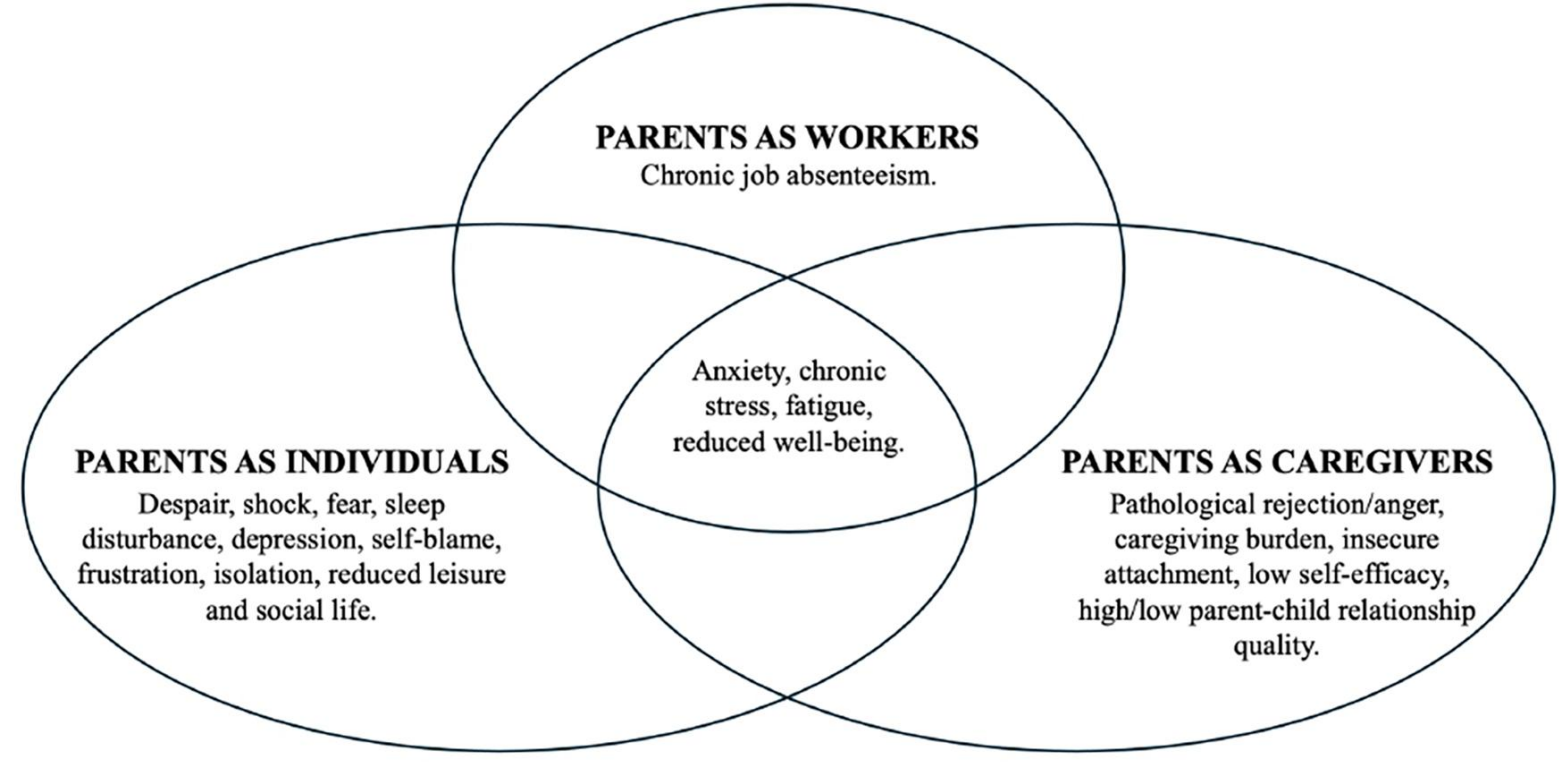
Summary of the main dimensions extracted based on the three identified focuses. In addition, the intersection shows the relationships between the three focuses.

In sum, the Euler-Venn diagram (Figure 2) graphically shows the dimensions identified for each focus as well as the overlapped dimensions among the parental roles have been reported.

## 4 Discussion

Given that atopic dermatitis is a chronic disease with potentially lifelong consequences, it is important to consider it from a systemic perspective (64), as it not only affects children's physical and psychological well-being but also significantly impacts their caregivers' quality of life. In line with family systems theory (63), the current review was guided by the idea that individual functioning should be understood within the context of broader family dynamics. Consequently, a diagnosis of atopic dermatitis in a child should be conceived in light of its impact on the entire family system, particularly on parents who assume primary caregiving responsibilities. Based on this, the Super's model (24) highlights the multiplicity of life roles served by individuals and how these roles evolve and interact across different life contexts. In the context of a family with a child diagnosed with atopic dermatitis, parents are required to continuously adjust and renegotiate their roles in response to the new demands imposed by the diagnosis. In this process, their occupational, personal, and caregiving functioning both influence and are influenced by the overall functioning of the family system. The idealised image of the child imagined by parents often contrasts with the real child struggling with itching, skin lesions, and sleep disturbances (38, 59). As a result, parents must adapt to this new reality, undergoing both emotional and practical reorganisation that affects various aspects of their lives. Due to the lack of review synthesising the impact of AD on parents, the current integrative literature review extracted the dimensions explored in parents of children with AD, categorise them into specific focuses, and provided a comprehensive narrative analysis. Consistent with both theoretical frameworks abovementioned, the extracted dimensions were examined across three interconnected focuses: (1) parents as workers, (2) parents as individuals, and (3) parents as caregivers.

Regarding the first focus (i.e., parents as workers), evidence highlighted that AD heavily impacts parents' work functioning, increasing stress and making work-family balance particularly challenging. Fathers often report career disruptions due to caregiving responsibilities, while some parents modify their career paths or change jobs entirely.

On the second focus (i.e., parents as individuals), AD affects several dimensions of parents' well-being, including quality of life, family burden, stress, anxiety, depression, hopelessness, coping strategies, and sleep quality. These results hyperbolically increased the emotional (e.g., quality of life, stress, anxiety), physical (e.g., sleep deprivation), and financial challenges faced by parents.

Concerning the third focus (i.e., parents as caregivers), AD influenced parent-child relationships. Studies indicated low physical and emotional engagement, such as lower levels of eye contact and vocal communication (38), which may contribute to insecure attachment bonds (39). Nevertheless, some mothers perceive the increased caregiving demands as an opportunity to strengthen their bond with their child, fostering greater physical closeness and emotional intimacy (37).

### 4.1 Risk and protective factors in parental functioning and adaptation to a child's atopic dermatitis

Beyond the extracted dimensions, several risk and protective factors were identified. Key risk factors include the severity of AD, the child's age and sex, and parental sex. Greater AD severity is associated with disrupted sleep routines, financial strain due to high treatment and medication costs, increased parental irritability, and reduced capacity to manage daily caregiving tasks (35, 52, 65–70). These factors, in turn, negatively affect parents' quality of life and mental health.

On the child's sex and age, studies highlighted that families of girls with AD reported a lower quality of life compared to those of boys (71, 72), suggesting sex-based differences in how families experience AD. Younger children necessitate more intensive care, increasing parental burden (73). However, in line with the study by Jirákova et al. (74), it is also important to focus on adolescence, given the impact on quality of life at this developmental stage. Parental sex also plays a pivotal role. Mothers are more culturally likely to assume primary caregiving responsibilities, leading to greater household management burdens (20) and challenges in balancing professional and family duties, particularly in full-time employment (40). Studies indicate that mothers are more vulnerable to stress than fathers and face a higher risk of suicidal ideation (16).

On protective factors, evidence reported that the higher the parental educational levels and the shorter the disease duration, the higher the quality of life (75). Furthermore, cohesive and flexible family functioning supports adaptation to caregiving demands (40). Letourneau et al. (76) highlighted the protective role of maternal sensitivity and social support, while excessive maternal directiveness and low responsiveness are linked to an increased risk of AD. In addition, connections with other parents of children with eczema were perceived as helpful in reducing the caregiving burden (44).

These results underscore the dynamic and processual nature of the parental journey following the communication of the child's AD diagnosis (77). Initially, parents often experience emotional shock, followed by phases of distress, frustration, and exhaustion. These emotional responses influence both parental individual functioning (e.g., particularly through sleep deprivation and heightened stress) and the quality of the parent-child relationship, which can become strained (63, 78, 79).

This trajectory aligns with previous research on parents of children with chronic diseases and long-term disabilities. Receiving a diagnosis of a chronic disease requires parents to undergo profound psychological and emotional adjustments, often involving a reorganisation of daily life, a redefinition of family roles, and adaptations in work-life balance, finances, and social relationships (62). Over time, parents may gradually accept their child's diagnosis, develop adaptive coping strategies, and reconstruct their expectations. This cognitive and emotional restructuring represents the most functional response to chronic disease. Reaching a stage of acceptance enables parents to effectively support their child's emotional needs, fostering emotional self-regulation (80) and secure attachment bond (63).

In sum, in line with the two theoretical frameworks guiding this integrative review (i.e., family systems theory and Super's model), the adaptive processes resulting from the parental ability to reorganise their multiple life roles show how changes in one member's functioning affect the entire family system, promoting overall family adaptation to the child's condition.

### 4.2 Policy implications and future direction

The present study aimed to examine how the child's atopic dermatitis affects parental functioning. To address this purpose, an integrative literature review was conducted to summarise the existing empirical literature and interpret it through well-established theoretical frameworks, and generate new policy insights and outline future directions.

Regarding policy implications, the review aims to provide information to professionals who support families of children diagnosed with AD to improve both their emotional well-being and the quality of care. Many parents express dissatisfaction with healthcare providers, feeling that their concerns are often overlooked or that medical professionals fail to fully address their child's needs (45, 47). Although AD is not life-threatening, its chronic and unpredictable nature develops ongoing challenges, particularly as parents must manage flare-ups and long-term treatment plans. As research on family adaptation to chronic disease highlights, the way a diagnosis is communicated can significantly impact the family's emotional response and coping strategies (81).

In the case of AD, parents often experience initial shock and uncertainty, which may be compounded by the fluctuating course of the disease and difficulties in treatment management (82). Structured support from healthcare professionals is critical at this stage, helping parents navigate the emotional and practical aspects of the diagnosis. Clear, realistic information about the chronic nature of AD, along with guidance on treatment expectations, is essential to reducing frustration and feelings of guilt (83). Many parents report a need for non-judgmental, empathetic support that reassures them about the efficacy of treatment and helps them manage their expectations (84).

Effective communication of the diagnosis should be multifaceted, including not only medical explanations but also emotional support and guidance for parents. This approach can empower parents by helping them feel more in control of their child's disease. Involving both parents in the care process is also crucial, as shared responsibility can reduce the burden on one caregiver. Additionally, healthcare providers' empathy, professionalism, and attentiveness strongly influence parental satisfaction and adherence to treatment plans (85). When parents establish a trusting relationship with healthcare providers, they feel heard and supported. This sense of trust toward professionals encourages them to engage more positively with disease management and maintain a strong connection to the healthcare system. As a result, treatment adherence is likely to improve for both the parent (86–88) and the child, as early experiences of trust can shape lifelong patterns, influencing the child's long-term treatment adherence (89–92).

Regarding the clinical setting, the review's findings paved the way for reflections on public policies: For instance, the design of evidence-based guidelines considering the family-centred communication for paediatric chronic conditions, such as AD, may be promoted; in addition, allocating professional resources to integrated psychological support services that consider parents' multiple roles would ensure that families receive not only medical care but also psychological and educational assistance throughout the disease trajectory. Furthermore, AD-focused training programmes could enhance healthcare professionals' and empathetic skills. In this vein, Figure 2 summarises the main dimensions affected by having a child with AD and on which training protocol may be designed. Finally, policies that acknowledge the long-term burden of AD on families (e.g., by providing access to counselling services, support networks) would contribute to improving families' overall quality of life.

Regarding future direction in research, longitudinal studies could be designed to explore how family experiences and relational dynamics change over time, e.g., from the time of receiving the diagnosis and through different children's developmental stages (i.e., childhood, adolescence, adulthood). In addition, future studies could benefit from adopting more standardised methodologies and measurement tools, which may enable the use of quantitative synthesis approaches to estimate the impact of childhood AD on parental functioning. Also, mixed-method approaches could be designed to capture both the prevalence and the nuanced meanings of parental experiences. In addition, future research should adopt a more systemic perspective, considering the experiences of all family members to better understand family dynamics as a whole (93–102). The conceptualisation og the Greater Patient by Basra & Finlay's (103) supports this approach: The authors highlighted that the person affected by a skin disease is part of a close social and familial network that also undergoes the burden of the condition.

Future studies could be designed to deepen the outcome roles played by the extracted dimensions, but also investigate their predictor or moderator role on the quality parent-child relationship.

Given the crucial role of healthcare professionals in managing chronic diseases, further studies should also examine the parent-physician-child relationship and its impact on treatment adherence. Another important area for investigation could be the acceptance of the diagnosis: studying how parents of children with AD react to and eventually accept the diagnosis could inform interventions aimed at facilitating this process within the family. Conceptualising acceptance of diagnosis as a psychological resource could offer new perspectives on how parents cope with caregiving demands, ultimately improving family well-being.

Finally, in terms of intervention, targeted programmes could help reduce the emotional burden on parents, enhancing both their mental health and their ability to support their child. Addressing these aspects could contribute to the development of more effective policies and support systems for families managing chronic diseases such as atopic dermatitis.

### 4.3 Limitations and conclusion

The current study highlighted the need to shift the focus from the individual patient to the entire family system in the context of an atopic dermatitis diagnosis. While existing studies provided valuable insights into the parental experience, some limitations persist. Most studies are cross-sectional, without capturing how experiences evolve. Additionally, research has largely focused on mothers, who are typically the primary caregivers, leaving the experiences of fathers and typically developing siblings underexplored. Taken together, these limitations point to a broader issue: understanding parental functioning in the context of atopic dermatitis requires a more comprehensive and systemic approach. By recognising the gaps in existing evidence, the present integrative review underscores the interdependent nature of family members' experiences and the importance of considering the whole family system. The management of AD affects each individual within the family, with roles and responsibilities that are deeply interconnected. For this reason, future research and clinical practice should adopt systemic frameworks that more fully capture the dynamic interplay between family members as they navigate the challenges of AD together.
