# Supplementary material for: When a child lives with atopic dermatitis: an integrative literature review on parental experience
Source: Front Pediatr. 2025 Dec 10;13:1720595. doi: 10.3389/fped.2025.1720595 (PMC12727998; doi:10.3389/fped.2025.1720595)
Supplement: Supplementary file 1 [file Table1.docx]

Supplementary Material

TABLE 1. Overview of all included studies’ descriptive characteristics

| **Dimension** | **Author(s), year** | **Country**  **(Global North *vs.* Global South)** | | **Study design (cross-sectional *vs.* longitudinal); Research method (quantitative *vs.* qualitative *vs.* mixed)** | | **Participants characteristics (sample size; sex distribution; mean age and standard deviation; age range)** | **Measure(s) and the dimensions evaluated** | **Aim(s) of the study** | **Main Results** |
| --- | --- | --- | --- | --- | --- | --- | --- | --- | --- |
| *Parents as workers* | | | | | | | | | |
| Work absenteeism | Cheng et al., (26) | | USA  (Global North) | | Cross-sectional; quantitative study | *Clinical Groups*  *Group 1*  *n* = 3,132 parents of children/adolescents with AD (sex distribution: ns);  *M(SD)* = ns;  Age range = ns.  *n* = 3,132 children/adolescents with AD (sex distribution: ns);  *M(SD)* = ns;  Age range = 3-22 years.  *Group 2*  *n* = 200 parents of children/adolescents with psoriasis (sex distribution: ns);  *M(SD)* = ns;  Age range = ns.  *n* = 200 children/adolescents with psoriasis (sex distribution: ns);  *M(SD)* = ns;  Age range = 3-22 years.  *Control Group*  *n* = 120,935 parents of TD children/adolescents (sex distribution: ns);  *M(SD)* = ns;  Age range = ns.  *n* = 120,935 TD children/adolescents (sex distribution: ns);  *M(SD)* = ns;  Age range = 3-22 years. | *Ad hoc* questions: school and work absenteeism. | (a) To investigate the burden and predictors of chronic school absenteeism in children/adolescents with AD and of work absenteeism in their parents. | AD was associated with chronic school absenteeism. Parents of children/adolescents with AD were more likely to miss work for caregiving compared to parents of children without AD. |
| *Parents as individuals* | | | | | | | | | |
| Family Burden | Muzzulon et al., (46) | | Brazil  (Global South) | | Cross-sectional; qualitative study | *n* = 23 mothers of children with AD;  *M(SD)* = 33.1 (12) years;  Age range = 21-50 years.  *n* = 23 children with AD (sex distribution: ns);  *M(SD)* = ns;  Age range = 2-10 years. | *Ad hoc* semi-structured interview: emotional and social impact of AD;  SCORAD: AD severity. | (a) To evaluate the emotional and social impact of AD on mothers of AD children. | Mothers, faced with the diagnosis of AD, experienced feelings of "despair," "fear," and "shock." Managing the disease, along with the challenges of diagnosis and treatment, created significant emotional strain. They are usually the primary caregivers and describe the care as difficult due to the daily experience with the disease and the constant attention required for the child. |
| Family Burden | Chua et al., (44) | | Singapore (Global North) | | Cross-sectional; qualitative study | *n* = 14 parents of children/adolescents with AD (mothers: 78,57%);  *M(SD)* = ns;  Age range = 30-59 years.  *n* = 14 children/adolescents with AD (mothers: 50%);  *M(SD)* = ns;  Age range = 11-15 years. | *Ad hoc* semistructured interview: experience in relation to their children/adolescents’ AD;  SCORAD: AD severity. | (a) To examine the impact of childhood AD on parents. | Parents reported that their lives were significantly impacted by their child's eczema symptoms, with mothers often emphasising the child's physical pain, while fathers tended to normalise the symptoms despite their severity. Environmental and dietary factors were commonly identified as triggers. Care responsibilities included ensuring treatment adherence and managing the itch-scratch cycle, which contributed to parental fatigue. Parents also highlighted the importance of fostering self-discipline and good habits in their children. Support from spouses and connections with other parents of children with eczema were perceived as helpful in reducing the caregiving burden. |
| Family Burden | Capozza et al., (42) | | United States United Kingdom Canada Australia  Ireland  Croatia  Netherlands  South Africa  Zambia  Bosnia and Herzegovina Estonia  Hong Kong India Malaysia New Zealand Poland Singapore  (Global North & Global South) | | Cross-sectional; quantitative study | *n* = 235 parents of children with AD (sex distribution: ns);  *M(SD)* = ns;  Age range = ns.  *n* = 235 children with AD (females: 42.6%);  *M(SD)* = 5.10 (4.59) years;  Age range = ns. | CADIS: parent emotion, sleep, and concerns about child esteem and social isolation;  POEM: AD severity. | (a) To examine the impact of childhood AD on parents. | Parents frequently experienced sleep disturbances, exhaustion, worry, and social isolation because of their child's AD. |
| Family Burden | Cheung & Lee, (41) | | China  (Global North) | | Cross-sectional; qualitative study | *n* = 9 mothers of children/adolescents with AD;  *M(SD)* = ns;  Age range = 30-45 years.  Specifically:  *n* = 4 (30–35 years);  *M(SD)* = ns.  *n* = 4 (36–40 years);  *M(SD)* = ns.  *n* = 1 (41–45 years);  *M(SD)* = ns.  *n* = 9 children/adolescents with AD (sex distribution: ns);  *M(SD)* = ns;  Age range = 2-14 years. | *Ad hoc* semistructured interview: experience in relation to their children/adolescents’ AD. | (a) To explore mothers’ experiences of caring for their AD children/adolescents. | Mothers faced challenges in managing their children/adolescents’ AD without additional support from family or community. Caring required them to take on extra responsibilities, with some quitting their jobs and sacrificing personal goals, such as pregnancy, leisure, hobbies, and social life. These mothers became experts in managing the disease, seeking medical care, and researching treatments for their children’s symptoms. Their emotional well-being was significantly impacted, with guilt and self-blame being constant struggles. |
| Family Burden | Santer et al., (47) | | UK  (Global North) | | Cross-sectional; qualitative study | *n* = 28 parents of children with AD (mothers: 24; fathers: 1; both parents: 3);  *Median* =36 years;  *M(SD)* = ns;  Age range = 26-46 years.  *n* = 28 children with AD (sex distribution: ns);  *Median* = 3 years;  *M(SD)* = ns;  Age range = 7 months - 5 years. | *Ad hoc* semistructured interview: experience in relation to their child AD. | (a) To explore parents’ views of childhood AD. | Parents reported feelings of frustration regarding their child's AD. The condition disrupts daily activities and causes concerns about the future. There is also anxiety regarding the appropriate treatments to use. Some parents expressed that, at times, doctors do not consider the issue to be serious enough and provide insufficient support. |
| Family Burden | Chamlin et al., (43) | | USA  (Global North) | | Cross-sectional; qualitative study | *n* = 26 parents of children with AD (sex distribution: ns);  *M(SD)* = ns;  Age range = ns.  *n* = 26 children with AD (females: 38.46%);  *M(SD)* = 23 (ns) months;  Age range = 3–69 months. | *Ad hoc* focus sessions: how atopic dermatitis affected children with AD and their parents. | (a) To explore effects of childhood AD on young children and their families. | Sleep disruption and itching/scratching were the most common physical symptoms in AD. Emotionally, irritability, fussiness, and increased crying were frequently reported. Parents expressed significant emotional effects, feelings of guilt and self-blame. Parents also described negative experiences with extended family members, where critical comments led to doubts about their parenting skills. Concerned about potential aggravating factors, parents restricted their children from activities such as swimming and outdoor play. Parents reported feelings of blame, conflict, and lack of support from relatives and friends. |
| Family Burden | Elliot & Lucker, (45) | | UK  (Global North) | | Cross-sectional; qualitative study | *n* = 77 mothers of children with AD;  *M(SD)* = ns;  Age range = ns.  *n* = 77 children with AD (sex distribution: ns);  *M(SD)* = ns;  Age range = ns. | *Ad hoc* open question: mothers’ experience about caring for an AD child. | (a) To explore the effects of caring for an AD child on mothers’ quality of life. | Mothers describe caring for a child with AD as an added physical burden to their daily responsibilities. The constant care, lack of sleep, and limited personal time lead to distress. They struggle to ask for help and often face challenges in their relationship with their partner. |
| Quality of life | Mozyrska, (70) | | Ukraine  (Global South) | | Cross-sectional; quantitative study | *n* = 104 parents of children/adolescents with AD (mothers: 79.8%);  *M(SD)* = ns;  Age range = ns.  *n* = 104 children/adolescents with AD (females: 48.1%);  *M(SD)* = ns;  *Median* = 6 years;  Age range = 6 months - 17 years. | FDLQI: impact of the children’s AD on the parents’ quality of life;    SCORAD: AD severity. | (a) To assess the quality of life of the families of AD children;  (b) To study the correlation between the AD severity and the quality of life of the family. | Four parents reported no impact of the disease on the family’s quality of life, while 65 indicated a mild effect, and 34 reported a moderate impact. Across the entire sample, greater disease severity was associated with lower parental quality of life. Children's age and disease duration were not related to quality of life. |
| Quality of life | Barbarot et al., (53) | | North America, Latin America, Europe, Middle East/Eurasia, and East Asia  (Global North & Global South) | | Cross-sectional; quantitative study | *n* = 7,465 parents of children with AD;  *n* = 7,465 AD children.  Specifically:  *Group 1*  *n* = 1,489 parents of children with AD (mothers: 62.8%):  *M(SD)* = 32.1 (7.4) years;  Age range = ns.  *n* = 1,489 children with AD (females: 48.5%);  *M(SD)* = 3.0 (1.6) years;  Age range = 6 months < 6 years.  *Group 2*  *n* = 2,898 parents of children with AD (mothers: 55.2%);  *M(SD)* = 40.5 (8.4) years;  Age range = ns.  *n* = 2,898 children with AD (females: 51.3%);  *M(SD)* = 14.9 (1.7) years;  Age range = 6 years - 12 years. | DFI: impact of AD on quality of life of the family;  *Ad hoc* questions: AD-related care and missed work days;  POEM: AD severity;  PtGA: AD severity. | (a) To evaluate the impact of childhood AD on families. | AD severity had a greater impact on family quality of life, including effects on sleep and tiredness. AD severity was positively associated with time spent on childcare and missed workdays. |
| Quality of life | Köse et al., (104) | | Turkey  (Global South) | | Cross-sectional; quantitative study | *n* = 122 parents of children with AD (sex distribution: ns);  *M(SD)* = ns;  Age range = ns.  *n* = 122 children with AD (females: 45.9%);  *M(SD)* = 5.4 (2.3) months;  Age range = 2–11 months. | IDQL: child’s quality of life;  FDLQI: impact of the children’s AD on the parents’ quality of life;  SCORAD: AD severity.  EASI: AD severity. | (a) To investigate the relationship between the AD severity and the quality of life of children with AD and  their families;  (b) To examine the factors that predict the severity of AD and the quality of life. | There was a significant relationship between AD severity and the quality of life of both children with AD and their parents. The child’s quality of life emerged as the most significant predictor of the impact of the AD on the parents' quality of life. |
| Quality of life | Ozsaydi et al., (33) | | Turkey  (Global South) | | Cross-sectional; quantitative study | *Clinical group*  *n* = 83 parents of children with AD (mothers: 79.3%);  *M(SD)* = ns;  Age range = ns.  *n* = 83 children with AD Age range = 2–7 years.  Specifically:  *Sub-group 1*  *n* = ns (females: 38.2%);  *M(SD)* = 33.1 (7.7) months;  Age range = 2-4 years.  *Sub-group 2*  *n* = ns (females: 47.2%);  *M(SD)* = 64.8 (8.9) months.  Age range = 5-7 years.  *Control group*  *n* = 83 parents of TD children (mothers: 79.7%);  *M(SD)* = ns;  Age range = ns.  *n* = 83 TD children;  Age range = 2–7 years.  Specifically:  *Sub-group 3*  *n* = ns (females: 37.5%);  *M(SD)* = 36.7 (7.4) months.  Age range = 2-4 years.  *Sub-group 4*  *n* = ns (females: 60.5%);  *M(SD)* = 68.7 (10) months.  Age range = 5-7 years. | PedsQL: children’s quality of life;  FIS-DD: families’ quality of life;  PO-SCORAD: AD severity. | (a) To determine the quality of life in children with AD and their families;  (b) To investigate possible factors that might impact their quality of life. | Regarding the children's quality of life, no significant differences between the study and control groups were found. Parents of older children reported lower children's quality of life than parents of younger ones. Contradictory results have been highlighted: the greater the severity, the lower the children's quality of life but the higher the family's quality of life. Among children aged 2–4 years, the presence of comorbidities is associated with reduced family quality of life. Additionally, higher income is associated with better quality of life. |
| Quality of life | Pedersen et al., (105) | | Bangladesh (Global South) | | Cross-sectional; quantitative study | *n* = 2,242 parents of children (sex distribution: ns);  *M(SD)* = ns;  Age range = ns.  *n* = 2,242 children (females: 49.3%);  *M(SD)* = 28.8 (16.9) months;  Age range = 6-59 months.  *Sub-sample*:  *n* = 267 children with AD (sex distribution: ns);  *M(SD)* = ns;  Age range = 6-59 months. | IDQOL: health-related quality of life of children with AD below the age of four;  DFI: impact of AD on quality of life of the family;  ISAAC: AD prevalence/severity;  POEM: AD severity. | (a) To investigate psychosocial effects on childhood AD on children and their families. | The severity of AD was associated with a lower quality of life for both the children with AD and their family. |
| Quality of life | Ražnatović Djurović et al., (73) | | Montenegro (Global South) | | Cross-sectional; quantitative study | *n* = 200 parents children/adolescents with AD (mothers: 94%);  *M(SD)* = 38.75 (4.66) years;  Age range = ns.  *n* = 200 children/adolescents with AD (females: 61%);  *M(SD)* = 11.68 (2.73) years;  Age range = 5-16 years. | CDLQI: children’s health-related quality-of-life;  DFI: impact of AD on quality of life of the family;  SCORAD: AD severity. | (a) To evaluate the impact of AD on the quality of life of children/adolescents and their parents;  (b) To identify predictors affecting their quality of life. | Greater AD severity was associated with lower family quality of life. Better quality of life of the children with AD was associated to improved parental quality of life.  Poorer parental quality of life was associated with female sex, younger age of children/adolescents, higher AD severity, lower parental educational level, and the absence of a family history of AD. |
| Quality of life | Maksimovic et al., (75) | | Serbia  (Global South) | | Longitudinal; quantitative study | *n* = 80 parents of children with AD (sex distribution: ns);  *M(SD)* = 37.4 (5.5) years;  Age range = 21-49 years.  *n* = 80 children with AD (females: 44.9%).  *Group 1*  *M(SD)* = 38 (ns) months;  Age range = 0-4 years.  *Group 2*  *M(SD)* = 60 (ns) months;  Age range = > 4. | IDQOL: health-related quality of life of children with AD below the age of four;    CDLQI: health-related quality of life of children with AD > 4 years;  DFI: impact of AD on quality of life of the family;  SCORAD: AD severity. | (a) To examine factors associated with change in quality of life among parents of AD children. | Parental quality of life significantly improved after one year. Improvements were observed in fatigue/exhaustion, emotional distress, and the impact of involvement in the child's treatment.  Having an older AD child was associated with better parental quality of life after one year. Additionally, higher parental education levels, shorter AD duration, lower AD severity, and greater improvement in the child's quality of life during follow-up were associated with greater enhancements in parental quality of life over the one-year follow-up period. |
| Quality of life | Ražnatović Djurović et al., (106) | | Montenegro  (Global South) | | Cross-section and quantitative study | *n* = 186 parents of children with AD (sex distribution: ns);  *M(SD)* = ns;  Age range = ns.  *n* = 186 children with AD (females: 61.3%);  *M(SD)* = 2.35 (1.01) years;  Age range = 0-4 years. | IDQOL: health-related quality of life of children with AD below the age of four;  DFI: impact of AD on quality of life of the family;  SCORAD: AD severity. | (a) To evaluate the impact of childhood AD on the quality of life of children and their parents;  (b) To identify predictors of their quality of life. | Parents of children severe AD reported a poorer quality of life compared to parents of children with milder AD. No significant impact of the child’s sex on parental quality of life was found. Parents of children with AD accompanied by other atopic conditions experienced a lower quality of life. |
| Quality of life | Siafaka et al., (52) | | Greece  (Global North) | | Cross-section and quantitative study | *n* = 75 mothers of children with AD;  *M(SD)* = 34.19 (4.66) years;  Age range = 25-39 years.  *n* = 75 children with AD;  *M(SD)* = 33.06 (39.64) months;  Age range = 3-157 months.  Specifically:  *Group 1*  *n* = 54 children with AD (females: 33.3%);  *M(SD)* = 11.16 (9.35) months;  Age range = 3-42 months.  *Group 2*  *n* = 21 children with AD (females: 42.8%);  *M(SD)* = 89.38 (0.94) months;  Age range = 53-157 months. | Brief IPQ: individual’s perceptions concerning AD;  IDQOL: health-related quality of life of children with AD below the age of four;  CDLQI: health-related quality-of-life; of children with AD aged 4 to 16 years;  DFI: impact of AD on quality of life of the family;  SCORAD: AD severity. | (a) To investigate the quality of life of children with AD and their families;  (b) To assess how mothers' illness perceptions influence family quality of life. | Lower family quality of life was significantly associated with stronger beliefs about the burden of AD, greater fear of consequences on the child's life, higher levels of worry. AD was also reported to impact family finances and lead to increased time spent by mothers on childcare and additional household tasks. |
| Quality of life | Singh et al., (107) | | Montenegro  (Global South) | | Cross-sectional; quantitative study | *n* = 142 caregivers of children/adolescents with AD (mothers: 98%);  *M(SD)* = ns;  Age range = ns.  *n* = 142 children/adolescents with AD (females: 44%);  *M(SD)* = 5 (4) years;  Age range = ≤ 16. | DFI: impact of AD on quality of life of the family;  SCORAD: AD severity. | (a) To investigate factors affecting quality of life in caregivers of AD children/adolescents and to investigated the  associated effect of disease severity. | Greater severity of the AD was associated with lower family quality of life. Caregivers reported high levels of emotional distress, fatigue, and a reduction in family leisure activities. Involvement in treatment, food preparation and feeding, family sleep quality, and caregivers' relationships were moderately affected by AD severity. |
| Quality of life | Xu et al., (108) | | Singapore  (Global North) | | Cross-sectional; quantitative study | *n* = 559 caregivers of children/adolescents with AD (sex distribution: ns);  *M(SD)* = ns;  Age range = ns.  *n* = 559 children/adolescents with AD;  *M(SD)* = 6.61 (4.55) years;  Age range = < 16 years.  Specifically:  *n* = 292 male children/adolescents with AD;  *M(SD)* = 6.91 (4.72) years.  *n* = 292 female children/adolescents with AD;  *M(SD)* = 6.30 (4.36) years. | SF-36: caregivers’ health-related quality of life;  IDQOL: health-related quality of life of children with AD below the age of four;  CDLQI: health-related quality of life of children with AD aged from 4 to 16 years;  Electronic medical records: AD severity;  EASI: AD severity. | (a) To investigate health-related quality of life of AD children/adolescents and their caregivers. | When AD children/adolescents’ health-related quality of life declines, their caregivers' mental and physical health also tends to deteriorate. This effect is particularly pronounced among caregivers of children with severe AD. Additionally, older children/adolescents age appears to negatively affect caregivers' physical health. |
| Quality of life | Campos et al., (67) | | Brazil  (Global South) | | Cross-sectional; quantitative study | *n* = 51 parents of children/adolescents with AD (sex distribution: ns);  *M(SD)* = ns;  Age range = ns,  *n* = 51 children/adolescents with AD (females: 55%);  M(SD) = 9.5 (3.2) years;  Age range = 5-16 years. | IDQL: child’s quality of life;  DFI: impact of AD on quality of life of the family;  SCORAD: AD severity. | (a) To evaluate the impact of children/adolescents’ AD on children and their parents. | AD severity is associated with a lower quality of life for both children/adolescents with AD and their parents. |
| Quality of life | Marciniak et al., (20) | | Poland  (Global North) | | Cross-sectional; quantitative study | *n* = 96 parents of children with AD (50 mothers and 46 fathers; 4 patients’ fathers were not in contact with their family);  M(SD) = ns;  Age range = ns.  *n* = 50 children with AD (females: 28%);  M(SD) = 10.2 (6.5) months.  Age range = 2 - 24 months. | FDLQ: impact of the children’s AD on the parents’ quality of life;  IDQOL: quality of life of AD children;  SCORAD: AD severity.  EASI: AD severity. | (a) To evaluate the impact of childhood AD on children and their parents. | AD affected both parents' quality of life, with a stronger impact on mothers, who took on more caregiving and household responsibilities. For fathers, the influence was more pronounced in their work and education. Both parents reported significant financial strain due to their children's condition, along with negative effects on their emotional and physical well-being. |
| Quality of life | Chernyshov, (69) | | Ukraine, Czech Republic, Singapore, and Italy  (Global North & Global South) | | Cross-sectional; quantitative study | *n* = 167 parents of children with AD (sex distribution: ns)  *M(SD)* = ns;  Age range = ns.  n = 167 children with AD children (female: 55.09%)  *M(SD)* = ns;  Age range = 5-16 years.  Specifically:  Singapore Group  *n* = 35 children with AD (female: 42.86%);  *M(SD)* = 10.60 (2.56) years.  Czech Republic Group  *n* = 50 children with AD female: 62%);  *M(SD)* = 12.0 (3.73) years.  Italy Group  n = 48 children with AD (female: 50%);  *M(*SD) = 6.58 (1.93) years.  Ukraine Group  *n* = 44 children with AD (female: 61,36%);  *M(SD)* = 9.86 (2.91) years. | DFI: impact of AD on quality of life of the family;  CDLQI: children’s health-related quality-of-life;  SCORAD: AD severity. | (a) To investigate the impact of children’s AD on family quality of life; | The severity of AD was associated with lower quality of life. Parents of school-aged children with AD were generally less stressed, tired, and exhausted than parents of preschool-aged children. |
| Quality of life | Chernyshov, (68) | | Ukraine, Czech Republic, Singapore, the Netherlands, Brazil, and South Korea  (Global North & Global South) | | Cross-sectional; quantitative study | *n* = 419 parents of children with AD (sex distribution: ns);  *M(SD)* = ns;  Age range = ns.  *n* = 419 children with AD (sex distribution: ns);  *M(SD)* = ns;  Age range = 0-4 years | IDQL: child’s quality of life;  DFI: impact of AD on quality of life of the family; | (a) To compare the impact of AD on children and their families in different countries. | Parental quality of life correlated with children's quality of life across all countries. Emotional distress was a significant issue for most parents, except in Brazil. In all countries except Ukraine, a higher parental assessment of disease severity was linked to a greater impact of AD on family relationships. Korean parents faced more challenges with their child's treatment, while Dutch parents reported a lower impact on their quality of life. Singaporean patients experienced minimal impact of AD on their dressing. |
| Quality of life | Chernyshov, (72) | | Ukraine  (Global South) | | Cross-sectional; quantitative study | *n* = 50 parents of children with AD (sex distribution: ns);  M(SD) = ns;  Age range = ns.  *n* = 50 children with AD (females: 50%);  Specifically:  *Girls*  M(SD) = 21.40 (11.26) months;  Age range = 9–48 months.  *Boys*  M(SD) = 21.08 (10.48) months;  Age range = 10–48 months. | IDQL: child’s quality of life;  DFI: impact of AD on quality of life of the family;  SCORAD: AD severity. | (a) To investigate sex differences in health-related and family quality of life in families with AD children. | The impact of AD on both the child’s and the family’s quality of life was more pronounced in girls. Parents of girls more frequently noted that AD negatively affected their child’s mood and ability to enjoy family activities. In boys, older age was associated with a lower family quality of life, while in both groups, the child’s age correlated with increased parental emotional distress. Additionally, age impacted family relationships in families with girls only. |
| Quality of life | Do Amaral et al., (109) | | Brazil  (Global South) | | Cross-sectional; quantitative study | *n* = 50 parents of children/adolescents with AD (sex distribution: ns);  M(SD) = ns;  Age range = ns.  *n* = 50 children/adolescents with AD (females: 70%);  M(SD) = 11.3 (3.1) years;  Age range = 5-16 years. | IDQL: child’s quality of life;  DFI: impact of AD on quality of life of the family. | (a) To assess the impact of childhood AD on quality of life of children/adolescents and their family. | The primary symptoms reported included itching, dry skin, and lichenification.  Regarding the impact of children’s AD on family life, 30% of families reported a moderate impact, while 44% reported a high impact. |
| Quality of life | Jiràkova ́ et al., (74) | | Czech Republic  (Global North) | | Cross-sectional; quantitative study | *n* = 203 parents of children/adolescents with AD (mothers: 88.67%);  M(SD) = ns;  Age range = ns.  *n* = 203 children with AD.  Specifically:  *Group 1*  *n* = 120 children with AD (females: 51%);  M(SD) = 28.7 (20.8) months;  Age range = 2 months - 6 years.  *Group 2*  *n* = 48 children/adolescents with AD (females: 54%);  M(SD) = 9.7 (2) years;  Age range = 7-13 years.  *Group* 3  *n* = 35 adolescents with AD (females: 51.4%);  M(SD) = 15.4 (1.7) years;  Age range = 14–18 years. | IDQOL: health-related quality of life of children with AD below the age of six;    CDLQI-cartoon version: health-related quality of life of children with AD aged from 7 to 13 years;  CDLQI-text version: health-related quality of life of AD adolescents aged from 14 to 18 years;  DFI: impact of AD on quality of life of the family. | (a) To evaluate the quality of life of AD children/adolescents of different age groups and their families. | The impact of the children/adolescents’ AD on family quality of life was similar across the three age groups, with a slightly higher impact observed in the 14-18 age group. No differences emerged between mothers and fathers regarding the impact of the child's AD on family’s quality of life. |
| Quality of life | Al Robaee & Shahzad, (65) | | Saudi Arabia  (Global South) | | Cross-sectional; quantitative study | *n* = 774 parents of children with AD (sex distribution: ns);  M(SD) = ns;  Age range = ns.  *n* = 774 children with AD (females: 43.3%);  M(SD) = 65.84 (32.87) months;  Age range = 17-130 months. | DFI: impact of AD on family functioning;  SCORAD: AD severity. | (a) To assess the quality of life of parents of AD children;  (b) To explore association between parents’ quality of life and AD severity. | Parents of children with severe AD reported a lower quality of life compared to parents of children with mild or moderate AD. The age of the children with AD did not significantly impact the quality of life of their parents. The greatest impact was observed in domains related to the costs associated with managing the child’s AD and the disrupted sleep of family members. |
| Quality of life | Al Shobaili, (71) | | Saudi Arabia  (Global South) | | Cross-sectional; quantitative study | *n* = 447 caregivers of children with AD (sex distribution: ns);  *M(SD)* = ns;  Age range = ns.  *n* = 447 children with AD (females: 43%);  *M(SD)* = 65.9 (32.9) months;  Age range = 17-130 months. | DFI: impact of AD on family functioning;  SCORAD: AD severity. | (a) To investigate the impact of children’s AD on family life;  (b) To investigate the association between the severity of AD and family life. | Quality of life was moderately affected in 66.4% of cases and severely impacted in 6.9%. Results revealed a significant association between the AD severity and the degree of impairment in quality of life, with more severe disease correlating with greater disturbance in quality of life. Families of girls with AD reported lower quality of life compared to families of boys with AD. |
| Quality of life | Ricci et al., (35) | | Italy  (Global North) | | Cross-sectional; quantitative study | *Clinical group*  *n* = 45 parents of children with AD (sex distribution: ns);  M(SD) = ns;  Age range = ns.  *n* = 45 children with AD (sex distribution: ns);  M(SD) = 24 (ns) months;  Age range = 3–84 months.  *Control group*  *n* = 20 parents of TD children (sex distribution: ns);  M(SD) = ns;  Age range = ns.  *n* = 20 TD children (sex distribution: ns);  M(SD) = 24 (ns) months;  Age range = 3–84 months. | IDQL: child’s quality of life;  DFI: impact of AD on quality of life of the family.  SCORAD: AD severity. | (a) To determine the ways in which childhood AD affects the quality of life of children and their families. | Children with AD experienced slight to moderate impairments in quality of life compared to the control group, with itching, sleep disturbances, and mood changes being the most discomforting symptoms. Family quality of life was also moderately affected, with sleep disruptions, the economic burden of managing the condition, and parent fatigue and irritability being the main issues. |
| Quality of life | Balkrishnan  et al., (66) | | USA (Global North) | | Cross-sectional; quantitative study | *n* = 49 parents of children with AD (mothers: 90%);  *M(sd)* = 30.3 (8.1) years;  Age range = 15 - 57 years.  *n* = 49 children with AD (sex distribution: ns);  *M(sd)* = 4.7 (3.4) years;  Age range = 1 - 12 years. | Modified version DFI: impact of AD on quality of life of the family. | (a) To investigate the impact of children’s AD on family quality of life; | Lower family quality of life was associated with greater severity of AD and financial concerns about the child’s condition |
| Quality of life | Ben-Gashir et al., (110) | | UK  (Global North) | | Longitudinal; quantitative study | *n* = 106 caregivers of children with AD (mothers: 99%);  *M(SD)* = ns;  Age range = ns.  *n* = 106 children with AD (females: 50%);  *M(SD)* = 8.2 (ns) years;  Age range = 5-10 years. | DFI: impact of AD on quality of life of the family;  SCORAD: AD severity. | (a) To investigate the relationship between the quality of family life and the severity of the children AD. | Quality of family life was related to the severity of AD in children at baseline and 6 months later. |
| Quality of life;  Additional dimensions: distress, sleep quality. | Kobusiewicz, Tarkowski, Kaszuba, Lesiak et al., (31) | | Poland  (Global North) | | Cross-sectional; quantitative study | *Clinical group*  *n* = 88 mothers of children with AD;  *M(SD)* = 35.05 (6.56) years;  Age range = 19-52 years.  *n* = 88 children with AD (females: 32.95%);  *M(SD)* = 60.16 (56.60) months;  Age range = 1–228 months.  *Control group*  *n* = 52 mothers of TD children;  *M(SD)* = 34.81 (3.88) years;  Age range = 27-42 years.  *n* = 52 TD children (females: 44.23%);  *M(SD)* = 60.25 (37.71) months;  Age range = 5-132 months. | *Ad hoc* questions child’s itch intensity, onset, and AD duration;  FDLQI: impact of the children’s AD on the parents’ quality of life;  PSS-10: mothers’ perceived stress levels;  HADS: mothers’ anxiety and depression symptoms;  AIS: mothers’ sleep disturbances;  SCORAD: AD severity. | (a) To compare perceived stress levels, sleep patterns, depression, and anxiety in mothers of children with AD;  (b) To investigate the relationship between AD and the psychosocial functioning of mothers of AD children. | Higher AD severity correlates with impaired quality of life and increased stress in mothers; AD severity worsens sleep in mothers and children; AD severity and itch are linked to poorer quality of life, insomnia, and stress; mothers of children with AD reported higher anxiety and depression. |
| Quality of life;  Additional dimension: sleep quality. | Neri et al., (32) | | Italy  (Global North) | | Cross-sectional; quantitative study | *Clinical Group*  *n* = 160 parents of children/pre-adolescent with AD (sex distribution: ns);  *M(SD)* = ns;  Age range = ns.  *n* = 160 children/pre-adolescent with AD (females: 25%);  *M(SD)* = 8.8 (ns) years;  Age range = 6-11 years.  *Control group*  *n* = 100 parents of TD children/pre-adolescent (sex distribution: ns);  *M(SD)* = ns;  Age range = ns.  *n* = 100 TD children/pre-adolescent (females: 53%);  *M(SD)* = 8.3 (ns) years;  Age range = 6-11 years. | *Ad hoc* questions: emotional and physical impact of AD on children/pre-adolescent and their caregivers, school participation, presenteeism, bullying. | (a) To investigate the psychosocial impact of AD on Italian children/pre-adolescent (6–11 years old), and their caregivers. | AD significantly affects sleep quality for both children/pre-adolescent and parents. Parents often feel overwhelmed by the responsibility of parenting, with 1 in 10 needing to change jobs or alter their career paths. The emotional and personal well-being of parents is also greatly affected by the condition. AD children/pre-adolescent frequently experience negative emotions such as shame and worry, while reporting positive feelings like calmness, determination, and freedom less often than their peers without AD. They find it difficult to feel comfortable with their condition. AD children/pre-adolescent are more likely to be victims of bullying compared to control group. |
| Quality of life;  Additional dimension: distress. | Suresh et al., (111) | | South Africa  (Global South) | | Cross-sectional; quantitative study | *n* = 54 caregivers of children with AD (mothers: 94.4%);  *M(SD)* = ns;  *Median* = 30 years;  Age range = 26–34.5 years.  *n* = 54 children with AD (sex distribution: ns);  *M(SD)* = ns;  *Median* = 5 years;  Age range = 2.75–7 years. | DFI: impact of AD on quality of life of the family;  K10: caregivers’ symptoms of anxiety and depression;  SCORAD: AD severity. | (a) To explore the relationship between children AD and the quality of life and mental health of their caregivers. | Having a child with AD had a moderately negative effect on their quality of life, with the majority experiencing mild psychological distress. No correlation was found between psychological distress, quality of life, and AD severity. However, family quality of life was associated with caregivers' mental health. |
| Quality of life;  Additional dimension: health care costs. | Olsson et al., (112) | | Singapore  (Global North) | | Cross-sectional; quantitative study | *n* = 559 parents of children with AD (sex distribution: ns);  *M(SD)* = ns;  Age range = ns.  *n* = 559 children with AD.  Specifically:  *Group 1*  *n* = 199 children with AD (females: 51.8%);  *M(SD)* = 1.86 (1.22) years;  Age range = < 4 years.  *Group 2*  *n* = 360 children with AD (females: 48.3%);  *M(SD)* = 9.24 (3.46) years;  Age range = 4 -16 years. | IDQOL: health-related quality of life of children with AD below the age of four;  CDLQI: health related to physical and mental aspects of AD of children >4 years;  SF-36: caregivers’ health-related quality of life. | (a) To explore associations between health-related quality of life, health care costs, and cost-accelerating variables. | Healthcare costs for children with AD were influenced by disease severity, duration, and impact on quality of life. Higher costs were associated with a negative impact on the child’s personal relationships and poorer physical health in caregivers.  In older children, however, only AD severity emerged as a determining factor. In infants, the child’s mood also contributed to increased healthcare costs. |
| Quality of life;  Additional dimension: obsessive compulsive disorder symptoms. | Gunduz et al., (29) | | Turkey  (Global South) | | Cross-sectional; quantitative study | *Clinical group*  *n* = 70 mothers of children with AD;  *M(SD)* = 29.3 (3.6) years;  Age range = ns.  *n* = 70 children with AD (females: 32.9%);  *M(SD)* = 9.7 (12.3) months;  Age range = ns.  *Control group*  *n* = 50 mothers of TD children;  *M(SD)* = 30.6 (3.7) years;  Age range = ns.  *n* = 50 TD children (females: 50%);  *M(SD)* = 12.4 (9.2) months;  Age range = ns. | SF-36: mothers’ quality of life;  MOCI: mothers’ obsessive-compulsive symptoms;  SCORAD: AD severity. | (a) To investigate the effect of atopic dermatitis on quality of life in terms of maternal health and maternal obsessive-compulsive symptoms. | The results showed that having an AD child do not influence their mothers in terms of obsessive-compulsive symptoms. AD mothers reported poor physical functioning compared to mothers of TD children. |
| Quality of life;  Additional dimension: physical and mental health. | Ho et al., (51) | | Singapore  (Global North) | | Cross-sectional; quantitative study | *No family impact*  *n* = 51 mothers of children with AD;  *M(SD)* = 38.57 (ns) years;  Age range = ns.  *n* = 51 children with AD (females: 37.3%);  *M(SD)* = 6.88 (ns) years;  Age range = ns.  *Low family impact*  *n* = 28 mothers of children with AD;  *M(SD)* = 36.54 (ns) years;  Age range = ns.  *n* = 28 children with AD (females: 35.7%);  *M(SD)* = 6.56 (ns) years;  Age range = ns.  *Moderate family impact*  *n* = 19 mothers of children with AD;  *M(SD)* = 34.63 (ns) years;  Age range = ns.  *n* = 19 children with AD (females: 68.4%);  *M(SD)* = 4.84 (ns) years;  Age range = ns.  *High family impact*  *n* = 6 mothers of children with AD;  *M(SD)* = 36.60 (ns) years;  Age range = ns.  *n* = 6 children with AD (females: 50%);  *M(SD)* = 6.35 (ns) years;  Age range = ns. | IDQOL/ CDLQI: children’s health-related quality-of-life;  DFI: impact of AD on quality of life of the family;  SF-12. mothers’ physical and mental health;  SCORAD: AD severity. | (a) To investigates maternal physical and mental health. | Mothers reported the most significant difficulties in performing moderate activities like housework, a reduced sense of accomplishment due to poor physical or emotional well-being, and a decline in social interactions. |
| Stress;  Additional dimension: quality of life. | Lee et al., (16) | | South  (Global North) | | Cross-sectional; quantitative study | *Clinical Group*  *n* = 970 parents of children with AD.  Specifically:  *Fathers*  *M(SD)* = 41.26 (0.31) years;  Age range = ns.  *Mothers*  *M(SD)* = 38.9 (0.27) years;  Age range = ns.  *n* = 970 children with AD (females: 48,51%);  *M(SD)* = 9.75 (0.21) years;  Age range = ns.  *Control Group*  *n* = 5,933 parents of TD children  Specifically:  *Fathers*  *M(SD)* = 42.72 (0.15) years;  Age range = ns.  *Mothers*  *M(SD)* = 39.77 (0.12) years;  Age range = ns.  *n* = 5,933 TD children (females: 46,64%);  *M(SD)* = 10.48 (0.11) years;  Age range = ns. | *Ad hoc* questions: parents’ experienced stress;  EQ-5D: parents’ quality of life;  EQ-VAS: parents’ quality of life. | (a) To explore the psychological stress experienced by parents of AD children. | Mothers of children with AD were more likely to experience heightened stress and report suicidal thoughts compared to mothers of TD. Fathers did not show significant differences in stress levels or mental health outcomes, regardless of whether their children had AD. |
| Stress;  Additional dimensions: quality of life, family functioning. | Yamaguchi et al., (40) | | Japan  (Global North) | | Cross-sectional; quantitative study | *n* = 216 mothers of children with AD;  *M(SD)* = 36.0 (4) years;  Age range = ns.  *n* = 216 children (females: 40.75);  Age range = 2-6 years.  Specifically:  *n* = 49 (2 years old children with AD);  *n* = 52 (3 years old children with AD);  *n* = 43 (4 years old children with AD);  *n* = 37 (5 years old children with AD);  *n* = 35 (6 years old children with AD). | PSI-SF: parenting stress;  JCMV-CADIS: quality of life in families of children with AD;  FAI: family functioning;  SCORAD: AD severity. | (a) To examined how maternal parenting stress is affected by demographic background, children's AD, and their family systems. | The severity of AD and allergy-related complications in children did not significantly influence the parenting stress experienced by mothers of AD children. Instead, lower family cohesion and family system flexibility predicted higher parenting stress. Full-time work by mothers predicted lower parenting stress. |
| Stress;  Additional dimensions: satisfaction with life, depression, social support. | Gieler et al., (28) | | Germany  (Global North) | | Cross-sectional; quantitative study | *Group 1 (Single mother, child with AD)*  *n* = 16 mothers of children with AD;  *M(SD)* = 33.0 (4.13) years;  Age range = ns.  *n* = 16 children with AD (sex distribution: ns);  *M(SD)* = 4.17 (1.56) years;  Age range = ns.  *Group 2 (Mother with partner, child with AD)*  *n* = 32 mothers of children with AD;  *M(SD)* = 31.94 (7.02) years;  Age range = ns.  *n* = 32 children with AD (sex distribution: ns);  *M(SD)* = 4.26 (1.78) years;  Age range = ns.  *Group 3 (Single mother, TD child)*  *n* = 16 mothers of TD children;  *M(SD)* = 33.31 (6.86) years;  Age range = ns.  *n* = 16 TD children (sex distribution: ns);  *M(SD)* = 4.65 (1.23) years;  Age range = ns.  *Group 4 (Mother with partner, TD child)*  *n* = 32 mothers of TD children;  *M(SD)* = 34.5 (5.19) years;  Age range = ns.  *n* = 32 TD children (sex distribution: ns);  *M(SD)* = 4.34 (1.23) years;  Age range = ns. | KFB: mothers’ non-health-related stress;  FLZ: mothers’ satisfaction with life;  ADS: mothers’ depressive symptoms;  F-Sozu: individually-experienced social support/stress;  FEN: mothers’ specific problems concerning children’s AD. | (a) To examine whether single mothers of children with AD experience different levels of stress and coping challenges compared to mothers with partners. | Single mothers reported higher feelings of helplessness and aggression due to their child’s scratching behavior compared to partnered mothers of AD children. Single mothers of children with AD had the highest family stress levels and the lowest general life satisfaction. |
| Stress;  Additional dimensions: quality of life, family burden. | Warschburger et al., (57) | | Germany  (Global North) | | Cross-sectional; quantitative study | *n* = 187 parents of children with AD (mothers: 98%);  *M(SD)* = 30 (ns) years;  Age range = 20-44 years.  *n* = 187 children with AD (females: 39%);  Girls *M(SD)* = 4.25 (1.62) years;  Boys *M(SD)* = 3.44 (1.53) years;  Age range = 7 months - 7 years. | FEN: parents’ disease management and psychosocial stress;  SF-12: parents’ health-related quality of life;  IOF: parents’ perceived burden of caring for a chronically ill child;  SCORAD: AD severity. | (a) To evaluate the psychosocial well-being of parents of AD children;  (b) To explore the relationship between parental quality of life and disease-related as well as sociodemographic factors. | The study revealed high levels of psychological distress among parents. Parents of children with more severe AD reported a greater impact on family functioning, financial burden, and increased demands in disease management. The ability of parents to manage the disease was influenced by family circumstances, their well-being, and the severity of their child's AD. |
| Anxiety & Depression;  Additional dimension: sleep quality. | Moore et al., (18) | | UK  (Global North) | | Cross-sectional; quantitative study | *n* = 92 parents (55 mothers; 37 fathers) from 55 families.  *Group 1*  *n* = 26 parents of children with AD (sex distribution: ns);  *M(SD)* = ns;  Age range = ns.  *n* = 26 children with AD (females: 35%);  *M(SD)* = 3 (ns) years;  Age range = 2 - 6 years.  *Group 2*  *n* = 29 parents of children with asthma (sex distribution: ns);  *M(SD)* = ns;  Age range = ns.  *n* = 29 children with asthma (females: 24%);  *M(SD)* = 7 (ns) years;  Age range = 4 - 10 years. | HADS: parents’ feelings of anxiety and depression;  *Ad hoc* questions: parent’s sleep disturbance. | (a) To compare the impact of caring for a child with AD *vs.* asthma on parents' sleep and well-being. | Parents caring for children with AD reported significantly less sleep compared to those caring for children with asthma. Correlation was found between the severity of sleep disturbances and the levels of maternal anxiety and depression, as well as paternal anxiety. |
| Depression & Hopelessness;  Additional dimension: children temperament. | Pauli-Pott et al., (17) | | Germany  (Global North) | | Cross-sectional; quantitative study | *Cohort 1*  *Clinical Group*  *n* = 20 mothers of children with AD;  *M(SD)* = ns;  Age range = ns.  *n* = 20 children with AD (females: 50%);  *M(SD)* = ns;  Age range = 3-4 years.  *Control Group*  *n* = 20 mothers of TD children;  *M(SD)* = ns;  Age range = ns.  *n* = 20 TD children (females: 50%);  *M(SD)* = ns;  Age range = 3-4 years.  *Cohort 2*  *Clinical Group*  *n* = 20 mothers of children with AD;  *M(SD)* = ns;  Age range = ns.  *n* = 20 children with AD (females: 50%);  *M(SD)* = ns;  Age range = 10-12 months.  *Control Group*  *n* = 20 mothers of TD children;  *M(SD)* = ns;  Age range = ns.  *n* = 20 TD children (females: 50%);  *M(SD)* = ns;  Age range = 10-12 months. | EMKK - anxious overprotectiveness and rigidity subscales: maternal child-rearing attitudes;  Hopelessness scale: mothers’ depressive tendency and hopelessness;  IBQ: maternal perception of infant temperament. | (a) To investigate whether mothers of children with AD differ from mothers of TD children in terms of depressive symptoms, feelings of hopelessness, parenting attitudes, and their perception of their children' temperament. | Mothers of children with AD reported higher levels of depression and hopelessness, exhibited more anxiety and overprotectiveness, and perceived their infants as displaying fewer positive and more frequent negative emotional behaviors compared to mothers of TD children. |
| Coping;  Additional dimension: stress. | Kobusiewicz, Tarkowski, Kaszuba & Zalewska-Janowska, (30) | | Poland  (Global North) | | Cross-sectional; quantitative study | *Clinical group*  *n* = 88 mothers of children with AD;  *M(SD)* = 35.05 (6.56) years;  Age range = 19–52 years.  *n* = 88 children with AD (females: 32.96%);  *M(SD)* = 60.43 (56.60) months;  Age range = 1–228 months.  *Control group*  *n* = 57 mothers of TD children;  *M(SD)* = 34.81 (3.88) years;  Age range = 27–42 years.  *n* = 57 TD children (females = 44.23%);  *M(SD)* = 60.25 (37.71) months;  Age range = 5–132 months. | COPE: strategies for coping with stress;  PSS: perceived level of stress;  NRS: skin itch;  SCORAD: AD severity. | (a) To compare the coping strategies used by mothers of children with and without AD to manage stress.  (b) Explore the association between the coping strategies of mothers of children with AD and factors such as disease characteristics, stress levels, and quality of life. | No statistically significant differences in coping strategies were found between the groups (most frequent strategies: active coping, seeking social support for instrumental and emotional reasons, focusing on and venting of emotions, and planning). Higher level of stress promoted positive coping strategies in terms of reinterpretation and growth, focusing on and venting of emotions, and alcohol/drug disengagement. Quality of life impairment favored planning, suppression of competing activities, and turning to religion. |
| Quality of Sleep | Harbottle et al., (59) | | Canada  (Global North) | | Cross-sectional; mixed study | *n* = 32 mothers of children with AD;  *M(SD)* = 30.1 (4.2) years;  Age range = ns.  *n* = 32 children with AD (females = 40.6%);  *M(SD)* = 8.3 (4.6) months;  Age range = ns. | PASS: mothers’ sleep quality and quantity.  *Ad hoc* semi-structured interview: mothers’ sleep quality and quantity;  *Ad-hoc* questions: mothers’ sleep quality and quantity;  PO-SCORAD: AD severity. | (a) To examine maternal sleep disturbances related to AD;  (b) To assess the relationship between AD severity and infant sleep outcomes. | Mothers of children with moderate to severe AD reported higher children sleeplessness compared to those with mild AD. Regardless of AD severity, all mothers experienced sleep loss, primarily due to children itching and maternal worry. |
| Quality of Sleep | Forer et al., (27) | | Israel  (Global North) | | Cross-sectional; quantitative study | *Clinical Group*  *n* = 100 parents of children/adolescents with AD (mothers: 40%);  *M(SD)* = 39.32 (8.7) years;  Age range = 22-64 years.  *n* = 100 children/adolescents with AD (females: 56%);  *M(SD)* = 7.65 (4.4) years;  Age range = 2-16 years.  *Control Group*  *n* = 100 parents of TD children/adolescents (mothers: 60%);  *M(SD)* = 35.43 (7.7) years;  Age range = 18-64 years.  *n* = 100 TD children/adolescents (females: 41%);  *M(SD)* = 7.51 (4.8) years;  Age range = 2-18 years. | PSQI: parents’ sleep quality. | (a) To examine the impact of AD on parents' sleep quality. | Parents of AD reported longer sleep latency compared to the control group. Fathers of children with AD experienced more sleep disturbances than mothers. Parents in the control group reported greater daytime dysfunction than those in the AD group. Additionally, parents in the mild AD group reported shorter sleep duration than those in the moderate-severe AD and control groups. |
| Quality of Sleep | Ramirez et al., (34) | | UK  (Global North) | | Longitudinal; quantitative study | *Clinical Group*  *n* = 4,500 mothers of children with AD;  *M(SD)* = ns;  Age range = ns.  *n* = 4,500 children with AD (females: 51.3%);  *M(SD)* = ns;  Age range = 6 months – 11 years (10 times points).  *Control Group*  *n* = 5,599 mothers of TD children;  *M(SD)* = ns;  Age range = ns.  *n* = 5,599 TD children (females: 46%);  *M(SD)* = ns;  Age range = 6 months – 11 years (10 times points). | *Ad hoc* questions: time-varying measure of active AD, maternal sleep outcomes. | (a) To assess whether mothers of children with AD experience impaired sleep during the first 11 years of their child's life;  (b) To examine whether these sleep disturbances are associated with the severity of the child’s condition and the child’s own sleep problems. | Mothers of children with AD reported difficulty falling asleep, insufficient sleep, and daytime exhaustion throughout the first 11 years of childhood. |
| Quality of Sleep | Angelhoff et al., (113) | | Sweden  (Global North) | | Cross-sectional; qualitative study | *n* = 12 parents of children with AD (mothers: 91.67%);  *M(SD)* = ns;  Age range = 20 - 40 years.  *n* = 12 children with AD;  *M(SD)* = ns;  Age range = <2 years. | *Ad hoc* interview: quality of sleep. | (a) To explore parents' perceptions of sleep, the consequences of sleep loss, and strategies used to manage sleep loss. | Sleep loss negatively impacted parents' emotional state, mood, well-being, cognitive function, concentration, initiative, and sensitivity to stress and sound. Parents coped primarily by altering their behavior, establishing new routines, taking personal time, and seeking support from their partners. |
| Quality of Sleep | Meltzer & Booster, (36) | | USA  (Global North) | | Cross-sectional; quantitative study | *Study 1*  *Group 1*  *n* = 61 parents of children/adolescents with ventilator-assistance (mothers: 72%);  *M(SD)* = 43.50 (7.52) years;  Age range = 28-60 years.  *n* = 61 parents of children/adolescents with ventilator-assistance (females: 54%);  *M(SD)* = 3.74 (3.193) years  Age range = 4-19 years  *Group 2*  *n* = 63 parents of TD children/adolescents (mothers: 65%);  *M(SD)* = 10.55 (4.51) years;  Age range = 29-58 years.  *n* = 63 TD children/adolescents (females: 57%);  *M(SD)* = 10.02 (4.43) years;  Age range = 4-20 years.  *Study 2*  *Group 3*  *n* = 35 parents of children/adolescents with AD (mothers: 83%);  *M(SD)* = 34.63 (5.76) years;  Age range = 22-45 years.  *n* = 35 children/adolescents with AD (females: 60%);  *M(SD)* = 3.74 (3.193) years;  Age range = 1-13 years.  *Group 4*  *n* = 42 parents of children/adolescents with asthma (mothers: 85%);  *M(SD)* = 41.07 (8.02) years;  Age range = 23-53 years.  *n* = 42 children/adolescents with asthma (females: 56%);  *M(SD)* = 10.22 (5.13) years;  Age range = 1-17 years.  *Group 5*  *n* = 57 parents of children/adolescents with AD and asthma (mothers: 86%);  *M(SD) =* 38.53 (8.36) years;  Age range = 23-62 years.  *n* = 57 children/adolescents with AD and asthma (females: 65%);  *M(SD)* = 7.74 (5.33) years;  Age range = 1-19 years. | PSQI: parents’ sleep disturbance;  ISI: parents’ severity of problems with sleep;  *Ad hoc* questions: parents’ sleep disruption. | (a) To examine sleep patterns and disturbances in parents of children/adolescents with chronic illness. | Parents of children/adolescents with chronic illness reported poorer sleep quality, increased insomnia symptoms, and chronic partial sleep deprivation compared to healthy families. Caregivers of children/adolescents with ventilator assistance and AD experienced higher rates of sleep disruptions due to caregiving, while caregivers of children/adolescents with asthma reported more sleep disturbances related to stress about the child's health. |
| *Parents as caregivers* | | | | | | | | | |
| Parent-child Relationship | Batac et al., (37) | | Canada  (Global North) | | Cross-sectional; mixed methods study | *Clinical group*  *n* = 32 mothers of children with AD;  *M(SD)* = 31.3 (4.2) years;  Age range = ns.  *n* = 32 children with AD (females: 41%);  *M(SD)* = 8.2 (4.6) months;  Age range = ns.  *Control group*  *n* = 65 mothers of TD children;  *M(SD)* = 30.1 (4.2) years;  Age range = ns.  *n* = 65 TD children (females: 51%);  *M(SD)* = 7.2 (4.1) months;  Age range = ns. | PBQ: quality of a mother’s emotional attachment to their child;  PO-SCORAD: AD severity.  *Ad hoc* semi-structured interview: mother-child bond. | (a) To investigate the association between infantile atopic dermatitis and the maternal‐infant bond. | Mothers of children with AD reported higher scores on caregiving anxiety and pathological rejection/anger subscales compared to controls. Many mothers described the additional AD caregiving demands as a factor that strengthened their bond with the child, fostering physical intimacy and emotional closeness rather than resentment. |
| Parent-child Relationship & attachment | Cassibba et al., (39) | | Italy  (Global North) | | Cross-sectional; quantitative study | *n* = 40 mothers;  *M (SD)* = 32.52 (4.93);  Age range = 19–42 years.  *Clinical groups*  *Group 1*  *n* = 10 mothers of premature children;  *M(SD)* = ns;  Age range = ns.  *n* = 10 premature children (females: 60%);  *M(SD)* = 29 (2.71) weeks;  Age range = ns.  *Group 2*  *n* = 10 mothers of children with AD;  *M(SD)* = ns;  Age range = ns.  *n* = 10 children with AD (females: 60%);  *M(SD)* = ns;  Age range = ns.  *Control group*  *n* = 20 mothers of at-term and TD children;  *M(SD)* = ns;  Age range = ns.  *n* = 20 at-term and TD children (females: 60%);  *M(SD)* = ns;  Age range = ns. | AAI: adult state of mind with respect to the attachment;  SSP: quality of the attachment relationship with the mother;  EA: quality of emotional interaction between mother and child. | (a) To evaluate the associations between maternal attachment representations, emotional availability, and mother–child attachment in a clinical group (including premature children and AD children) and a control group. | The child's clinical condition did not affect maternal attachment representations or emotional availability. However, insecure attachment was more common in premature and AD infants. Mothers across groups showed similar attachment representations and sensitivity. In clinical dyads, Emotional Availability was linked to AAI but not to the Strange Situation. Regression analyses confirmed its predictive role for child security in the control group, but not in the clinical group. |
| Parent-child Relationship  Additional dimension: distress. | Letourneau et al., (76) | | Canada  (Global North) | | Longitudinal (early and late gestation, and postpartum); quantitative study | *n* = 242 mothers of children with AD;  *M(SD)* = 31.2 (3.80) years;  Age range = ns.  *n* = 242 children with AD (female = 49.59%)  Age = 18 months. | CARE: maternal–infant relationship quality;  EDS: maternal psychological distress;  SCL-90-R - anxiety subscale: maternal psychological distress;  PSAS: maternal psychological distress;  SLEQ: maternal psychological distress. | (a) To examine the association between maternal–infant relationship qualities (sensitivity, responsiveness, control) and child AD by age 2, considering risk (maternal depression, anxiety, stress) and protective (social support) factors;  (b) To test hypotheses that lower sensitivity, higher unresponsiveness/control, and increased maternal distress raise AD risk, while greater postnatal social support lowers it. | (a) High maternal sensitivity and social support, combined with low perinatal anxiety, are linked to a lower likelihood of childhood AD. In contrast, higher maternal control and unresponsiveness increase the odds of childhood AD.  (b) Poor maternal–infant relationship quality at 6 months (characterized by low sensitivity, high unresponsiveness, and high controlling behavior) increases the risk of childhood AD at 18 months. |
| Parent-child Relationship;  Additional dimensions: anxiety, depression. | Charfi et al., (38) | | Tunisia  (Global South) | | Cross-sectional; quantitative study | *n* = 48 mother-child dyads  *Clinical Group*  *n* = 24 mothers of children with AD;  *M(SD)* = 33.04 (5.85) years;  Age range = 23 – 42 years.  *n* = 24 children with AD (females: 58.33%);  *M(SD)* = 17.17 (8.70) months;  Age range = 5-36 months.  *Control Group*  *n* = 24 mothers of TD children;  *M(SD)* = 31.08 (5.75) years;  Age range = 19-45 years.  *n* = 24 TD children (females: 58.33%);  *M(SD)* = 16.08 (9.01) months;  Age range = 5-36 months. | BDI: mothers’ depression;  HAM-A: mothers’ anxiety;  Video recording: mother-child interaction;  SCORAD: AD severity. | (a) To examine the differences in interaction between AD mother-child dyads and TD dyads;  (b) To explore anxiety and depression in mothers. | Mothers of children with AD showed significantly higher anxiety scores compared to control dyads, but no significant differences in depression was found. They also exhibited impaired skin-to-skin contact, reduced eye contact, and fewer vocalizations with their children than control dyads. Moreover, maternal anxiety was positively correlated with inadequate maternal attitudes toward the child and increased child distress behaviors. |
| Parent-child Relationship & Self-efficacy;  Additional dimensions: emotional and behavioral problems in children, distress, parents’ relationship quality. | Mitchell et al., (114) | | Australia  (Global North) | | Cross-sectional; quantitative study | *n* = 64 parents of children with AD (mothers = 90.6%);  *M(SD)* = 35.70 (7.97) years;  Age range = ns.  *n* = 64 children with AD (females: 35.9%);  *M(SD)* = 5.88 (3.21) years;  Age range = 2-12 years. | CEMQ: self-efficacy about child’s AD management;  ECBI: behaviour problems for children;  SDQ - Emotional Symptoms subscale: emotional problems in children;  DASS-21: depression, anxiety, and stress symptoms;  PSI-SF: parenting stress;  QMI: parents’ relationship quality and satisfaction;  PPC: interparental conflict over child-rearing issues;  Observation of a routine AD treatment session: parent-child interaction.  SCORAD: AD severity. | (a) To examine whether child, parent, and family characteristics influence parental self-efficacy and self-reported  AD management performance;  (b) To assess whether self-efficacy mediates the relationship between these characteristics and parental self-reported AD management;  (c) To observe parent behavior during AD management to explore links between characteristics, self-efficacy, and observed treatment competence;  (d) To hypothesize that children with severe AD exhibit greater emotional and behavioral problems than those with mild or moderate AD. | Parental self-efficacy was associated with disease severity, child behavior difficulties, parent depression and stress, parenting conflict, and relationship satisfaction.  Self-efficacy mediated the relationship between child behavior, parental education, and self-reported task performance.  Observations showed strong links between parents’ treatment competence, self-efficacy, outcome expectations, and self-reported task performance.  Children with more severe AD exhibited greater behavioral challenges. |

Notes: *M*: Mean Age; *SD*: Standard Deviation; NS: Not Specified; AD: Atopic Dermatitis; TD: typically developing.

**List of Acronyms, Full Names, and References of the Measures Reported in Table 1**

AAI: Adult Attachment Interview (115);

ADS: General Depression Scale (116)

AIS: Athens Insomnia Scale (117,118);

BDI: Beck Depression Inventory – short form (reference not reported);

Brief IPQ: Brief Illness Perception Questionnaire (119);

CADIS: Childhood Atopic Dermatitis Impact Scale (120);

CARE: Child-Adult Relationship Experimental Index (121);

CDLQI: Children’s Dermatology Life Quality Index (122,123);

CEMQ: Child Eczema Management Questionnaire (124)

COPE: Coping Orientations to Problems Experienced (125);

DASS-21: Depression Anxiety Stress Scales-21 item (126);

DFI: Dermatitis Family Impact questionnaire (127,128);

EA: Mother-child dyads Emotional Availability Scales (129);

EASI: Eczema Area and Severity Index (130);

ECBI: Eyberg Child Behaviour Inventory (131);

EDS: Edinburgh Depression Scale (132,133);

EMKK: Fragebogen zur Erhebung der Einstellungen von Müttern mit Kindern im Kleinst- kindalter (134);

EQ-5D: EuroQol-5 dimension index (reference not reported);

EQ-VAS: EuroVisual Analogue Scale (reference not reported);

FAI: Family Assessment Inventory (135);

FDLQI: Family Dermatology Life Quality Index (136–138);

FEN: Questionnaire for Parents of Children with Atopic Eczema (139);

FIS-DD: Family Impact Scale for Dermatological Diseases (140);

FLZ: Satisfaction with Life Questionnaire (141);

F-Sozu: Questionnaire on Social Support (142);

HADS: Hospital Anxiety and Depression Scale (143);

HAM-A: Hamilton Anxiety Rating Scale (144);

Hopelessness scale (145);

IBQ: Infant Behavior Questionnaire (146,147);

IDQOL: Infants Dermatitis Quality of Life Index (148);

IOF: Impact on family scale (149);

ISAAC: International Study of Asthma and Allergies in Childhood (150);

ISI: Insomnia Severity Index (151);

JCMV-CADIS: Japanese Culturally Modified Version of the Childhood Atopic Dermatitis Impact Scale (152);

K10: Kessler Psychological Distress Scale Questionnaire (153,154);

KFB: Short Stress Questionnaire (154);

MOCI: The Maudsley Obsessive Compulsive Inventory (155);

NRS: Numerical Rating Scale (118,156);

PASS: Perinatal Anxiety Screening Scale (reference not reported);

PBQ: Postpartum Bonding Questionnaire (157);

PedsQL: Pediatric Quality of Life Inventory (158);

POEM: Patient-Oriented Eczema Measure (159);

PO-SCORAD: Patient- Oriented SCORing Atopic Dermatitis index (160);

PPC: Parent Problem Checklist (161);

PSAS: Pregnancy-Specific Anxiety Scale (162)

PSI-SF: Parenting Stress Index-Short Form (163,164);

PSQI: Pittsburgh Sleep Quality Index (165,166);

PSS: Perceived Stress Scale (167);

PSS-10: Perceived Stress Scale (167);

PtGA: Patient Global Assessment (reference not reported);

QMI: Quality Marriage Index (168);

SCL-90-R: Symptom Checklist-90 item-Revised (169);

SCORAD: SCORing Atopic Dermatitis index (170);

SDQ: Strengths and Difficulties Questionnaire (171)

SF-36: Health-Related Quality of Life questionnaire (172,173);

SF-12: Short-Form Health Survey (174,175);

SLEQ: Stressful Life Events Questionnaire (176);

SSP: Child Strange Situation Procedure (177).

**References**

104. *Köse SŞ, Akelma Z, Özmen S. Severity of disease and the quality of life indexes in infants with atopic dermatitis. Allergol Immunopathol. (2022) 50(3):55–61. doi: 10.15586/aei.v50i3.556.

105. *Pedersen CJ, Uddin MJ, Saha SK, Darmstadt GL. Prevalence and psychosocial impact of atopic dermatitis in Bangladeshi children and families. PLoS One. (2021) 16(4):e0249824. doi: 10.1371/journal.pone.0249824.

106. *Ražnatović Djurović MR, Janković J, Ćirković A, Spirić VT, Maksimović N, Timotijević ZS, et al. Quality of life in infants with atopic dermatitis and their families. Postepy Dermatol Alergol. (2020) 37(1):66–72. doi: 10.5114/ada.2020.93385.

107. *Singh B, Thandar Y, Balakrishna Y, Mosam A. The quality of life of caregivers of children with atopic dermatitis in a South African setting. S Afr J Child Health. (2019) 13(2):63–68. doi: 10.7196/SAJCH.2019.v13i2.1544.

108. *Xu X, van Galen LS, Koh MJA, Bajpai R, Thng S, Yew YW, et al. Factors influencing quality of life in children with atopic dermatitis and their caregivers: a cross-sectional study. Sci Rep. (2019) 9(1):15990. doi: 10.1038/s41598-019-51129-5.

109. *Do Amaral CSF, March MDFBP, Sant’Anna CC. Quality of life in children and teenagers with atopic dermatitis. An Bras Dermatol. (2012) 87:717–723. doi: 10.1590/S0365-05962012000500008.

110. *Ben-Gashir MA, Seed PT, Hay RJ. Are quality of family life and disease severity related in childhood atopic dermatitis? J Eur Acad Dermatol Venereol. (2002) 16(5):455–462. doi: 10.1046/j.1468-3083.2002.00495.x.

111. *Suresh S, Kannenberg SM, Lachman A. Assessing the impact paediatric atopic dermatitis has on the mental health and quality of life of caregivers attending a tertiary hospital in Cape Town, South Africa. Curr Allergy Clin Immunol. (2023) 36(2):2–7.

112. *Olsson M, Bajpai R, Yew YW, Koh MJA, Thng S, Car J, et al. Associations between health-related quality of life and health care costs among children with atopic dermatitis and their caregivers: a cross-sectional study. Pediatr Dermatol. (2020) 37(2):284–293. doi: 10.1111/pde.14071.

113. *Angelhoff C, Askenteg H, Wikner U, Edéll-Gustafsson U. “To cope with everyday life, I need to sleep”: a phenomenographic study exploring sleep loss in parents of children with atopic dermatitis. J Pediatr Nurs. (2018) 43:e59–e65. doi: 10.1016/j.pedn.2018.07.005.

114. *Mitchell AE, Fraser JA, Ramsbotham J, Morawska A, Yates P. Childhood atopic dermatitis: a cross-sectional study of relationships between child and parent factors, atopic dermatitis management, and disease severity. Int J Nurs Stud. (2015) 52(1):216–228. doi: 10.1016/j.ijnurstu.2014.09.008.

115. Main M, Kaplan N, Cassidy J. Security in infancy, childhood, and adulthood: a move to the level of representation. Monogr Soc Res Child Dev. (1985) 50(1–2):66–104. doi: 10.2307/3333827.

116. Bailer M, Hautzinger M, Hofmeister D, Keller F. Allgemeine Depressionsskala (ADS). Göttingen: Hogrefe. (2012).

117. Fornal-Pawłowska M, Wołyńczyk-Gmaj D, Szelenberger W. Validation of the Polish version of the Athens Insomnia Scale. Psychiatr Pol. (2011) 45(2):211–221.

118. Ständer S, Augustin M, Reich A, Blome C, Ebata T, Phan NQ, et al. Pruritus assessment in clinical trials: consensus recommendations from the International Forum for the Study of Itch (IFSI) Special Interest Group Scoring Itch in Clinical Trials. Acta Derm Venereol. (2013) 93(5). doi: 10.2340/00015555-1620.

119. Broadbent E, Petrie KJ, Main J, Weinman J. The brief illness perception questionnaire. J Psychosom Res. (2006) 60(6):631–637. doi: 10.1016/j.jpsychores.2005.10.020.

120. Chamlin SL, Lai JS, Cella D, Frieden IJ, Williams ML, Mancini AJ, et al. Childhood Atopic Dermatitis Impact Scale: reliability, discriminative and concurrent validity, and responsiveness. Arch Dermatol. (2007) 143(6):768–772. doi: 10.1001/archderm.143.6.768.

121. Crittenden PM. CARE-Index: infants coding manual. Miami, FL: Family Relations Institute. (2010).

122. Lewis-Jones MS, Finlay AY. The Children’s Dermatology Life Quality Index (CDLQI): initial validation and practical use. Br J Dermatol. (1995) 132(6):942–949.

123. Salek MS, Jung S, Brincat-Ruffini LA, MacFarlane L, Lewis-Jones MS, Basra MKA, et al. Clinical experience and psychometric properties of the Children’s Dermatology Life Quality Index (CDLQI), 1995–2012. Br J Dermatol. (2013) 169(4):734–759.

124. Mitchell AE, Fraser JA. Parents’ self-efficacy, outcome expectations, and self-reported task performance when managing atopic dermatitis in children: instrument reliability and validity. Int J Nurs Stud. (2011) 48(2):215–226. doi: 10.1016/j.ijnurstu.2010.06.008.

125. Carver CS, Scheier MF, Weintraub JK. Assessing coping strategies: a theoretically based approach. J Pers Soc Psychol. (1989) 56(2):267–283. doi: 10.1037/0022-3514.56.2.267.

126. Lovibond PF, Lovibond SH. Depression anxiety and stress scales. Behav Res Ther. (1995). doi: 10.1037/t39835-000.

127. Dodington SR, Basra MKA, Finlay AY, Salek MS. The Dermatitis Family Impact questionnaire: a review of its measurement properties and clinical application. Br J Dermatol. (2013) 169(1):31–46. doi: 10.1111/bjd.12232.

128. Lawson V, Finlay A, Reid P, Owens R. The family impact of childhood atopic dermatitis: the Dermatitis Family Impact Questionnaire. Br J Dermatol. (1998) 138(1):107–113. doi: 10.1046/j.1365-2133.1998.02034.x.

129. Biringen Z, Brown D, Donaldson L, Green S, Krcmarik S, Lovas G. Adult Attachment Interview: linkages with dimensions of emotional availability for mothers and their pre-kindergarteners. Attach Hum Dev. (2000) 2(2):188–202. doi: 10.1080/14616730050085554.

130. Hanifin JM, Thurston M, Omoto M, Cherill R, Tofte SJ, Graeber M, et al. The eczema area and severity index (EASI): assessment of reliability in atopic dermatitis. Exp Dermatol. (2001) 10(1):11–18. doi: 10.1034/j.1600-0625.2001.100102.x.

131. Eyberg SM. Eyberg Child Behavior Inventory and Sutter-Eyberg Student Behavior Inventory–Revised: professional manual. Lutz, FL: Psychological Assessment Resources. (1999).

132. Cox JL, Holden JM, Sagovsky R. Detection of postnatal depression: development of the 10-item Edinburgh Postnatal Depression Scale. Br J Psychiatry. (1987) 150(6):782–786. doi: 10.1192/bjp.150.6.782.

133. Matthey S. Differentiating between transient and enduring distress on the Edinburgh Depression Scale within screening contexts. J Affect Disord. (2016) 196:252–258. doi: 10.1016/j.jad.2016.02.004.

134. Engfer A. Fragebogen zur Erhebung der Einstellungen von Müttern mit Kindern im Kleinstkindalter (Questionnaire to Assess the Attitudes of Mothers with Small Infants). Munich: Institute of Psychology, University of Munich. (1984).

135. Nishide T. Kazoku assessment inventory no sakusei. Constructing the family assessment inventory (FAI): measuring family functioning. Jpn J Fam Psychol. (1993) 7:53–65.

136. Sajedianfard S, Handjani F, Saki N, Heiran A. Family dermatology life quality index in patients with pemphigus vulgaris: a cross-sectional study. Indian J Dermatol Venereol Leprol. (2021) 87(3):375–378. doi: 10.4103/ijdvl.IJDVL_276_18.

137. Basra MKA, Sue-Ho R, Finlay AY. The Family Dermatology Life Quality Index: measuring the secondary impact of skin disease. Br J Dermatol. (2007) 156(3):528–538. doi: 10.1111/j.1365-2133.2006.07617.x.

138. Marciniak J, Reich A, Szepietowski J. The Family Dermatology Life Quality Index: formulation and validation of the Polish version. Forum Dermatol. (2016) 2(1):24–28.

139. Stangier U, Ehlers A, Gieler U. Fragebogen zur Bewältigung von Hautkrankheiten (FBH). Göttingen: Hogrefe, Verlag für Psychologie. (1996).

140. Turan E, Gürel MS, Erdemir AT, Yüksel Eİ. Development and preliminary validation of the dermatological family impact scale. Turkderm. (2014) 48:74–81. doi: 10.4274/turkderm.27167.

141. Fahrenberg J, Myrtek M, Schumacher J, Brähler E. Fragebogen zur Lebenszufriedenheit (FLZ) [Life Satisfaction Questionnaire]. Göttingen: Hogrefe. (2000).

142. Fydrich T, Geyer M, Hessel A, Sommer G, Brähler E. Social support questionnaire (F-SozU): norms of a representative sample. Diagnostica. (1999) 45(4):212–216.

143. Zigmond AS, Snaith RP. The Hospital Anxiety and Depression Scale. Acta Psychiatr Scand. (1983) 67(6):361–370. doi: 10.1111/j.1600-0447.1983.tb09716.x.

144. Hamilton M. Hamilton anxiety rating scale (HAM-A). Br J Med Psychol. (1959) 32:50–55.

145. Krampen G. Hoffnungslosigkeit bei stationären Patienten: ihre Messung durch einen Kurzfragebogen (H-Skala). Med Psychol. (1979) 5(1):39–49.

146. Rothbart MK. Measurement of temperament in infancy. Child Dev. (1981) 52(2):569–578. doi: 10.2307/1129176.

147. Rothbart MK. Longitudinal observation of infant temperament. Dev Psychol. (1986) 22(3):356–365. doi: 10.1037/0012-1649.22.3.356.

148. Lewis-Jones MS, Finlay AY, Dykes PJ. The Infants’ Dermatitis Quality of Life Index. Br J Dermatol. (2001) 144(1):104–110. doi: 10.1046/j.1365-2133.2001.03960.x.

149. Stein RE, Riessman CK. The development of an impact-on-family scale: preliminary findings. Med Care. (1980) 18(4):465–472.

150. Lule SA, Mpairwe H, Nampijja M, Akello F, Kabagenyi J, Namara B, et al. Life-course of atopy and allergy-related disease events in tropical sub-Saharan Africa: a birth cohort study. Pediatr Allergy Immunol. (2017) 28(4):377–383. doi: 10.1111/pai.12719.

151. Bastien CH, Vallières A, Morin CM. Validation of the Insomnia Severity Index as an outcome measure for insomnia research. Sleep Med. (2001) 2(4):297–307. doi: 10.1016/S1389-9457(00)00065-4.

152. Chamlin SL, Cella D, Frieden IJ, Williams ML, Mancini AJ, Lai JS, et al. Development of the Childhood Atopic Dermatitis Impact Scale: initial validation of a quality-of-life measure for young children with atopic dermatitis and their families. J Invest Dermatol. (2005) 125(6):1106–1111. doi: 10.1111/j.0022-202X.2005.23911.x.

153. Van Heyningen T, Honikman S, Tomlinson M, Field S, Myer L. Comparison of mental health screening tools for detecting antenatal depression and anxiety disorders in South African women. PLoS One. (2018) 13(4):e0193697. doi: 10.1371/journal.pone.0193697.

154. Flor H. Kurzer Fragebogen zur Erfassung von Belastungen. In: Psychobiologie des Schmerzes. Bern: Huber. (1991). p. 144–153.

155. Hodgson RJ, Rachman S. Obsessional-compulsive complaints. Behav Res Ther. (1977) 15(5):389–395. doi: 10.1016/0005-7967(77)90042-0.

156. Reich A, Heisig M, Phan NQ, Taneda K, Takamori K, Takeuchi S, et al. Visual analogue scale: evaluation of the instrument for the assessment of pruritus. Acta Derm Venereol. (2012) 92(5):497–501. doi: 10.2340/00015555-1265.

157. Brockington IF, Oates J, George S, Turner D, Vostanis P, Sullivan M, et al. A screening questionnaire for mother-infant bonding disorders. Arch Womens Ment Health. (2001) 3:133–140. doi: 10.1007/s007370170010.

158. Uneri OS, Agaoglu B, Coskun A, Memik NC. Validity and reliability of Pediatric Quality of Life Inventory for 2-to 4-year-old and 5-to 7-year-old Turkish children. Qual Life Res. (2008) 17:307–315. doi: 10.1007/s11136-007-9303-4.

159. Charman CR, Venn AJ, Williams HC. The patient-oriented eczema measure: development and initial validation of a new tool for measuring atopic eczema severity from the patients’ perspective. Arch Dermatol. (2004) 140(12):1513–1519. doi: 10.1001/archderm.140.12.1513.

160. Stalder JF, Barbarot S, Wollenberg A, Holm EA, De Raeve L, Seidenari S, et al. Patient-oriented SCORAD (PO-SCORAD): a new self-assessment scale in atopic dermatitis validated in Europe. Allergy. (2011) 66(8):1114–1121. doi: 10.1111/j.1398-9995.2011.02577.x.

161. Dadds MR, Powell MB. The relationship of interparental conflict and global marital adjustment to aggression, anxiety, and immaturity in aggressive and non-clinic children. J Abnorm Child Psychol. (1991) 19:553–567. doi: 10.1007/BF00925820.

162. Rini CK, Dunkel-Schetter C, Wadhwa PD, Sandman CA. Psychological adaptation and birth outcomes: the role of personal resources, stress, and sociocultural context in pregnancy. Health Psychol. (1999) 18(4):333–341.

163. Abidin RR. Parenting Stress Index–Short Form. Charlottesville, VA: Pediatric Psychology Press. (1990).

164. Abidin RR. Parenting Stress Index. 3rd edn. Lutz, FL: Psychological Assessment Resources. (1995).

165. Buysse DJ, Reynolds CF III, Monk TH, Berman SR, Kupfer DJ. The Pittsburgh Sleep Quality Index: a new instrument for psychiatric practice and research. Psychiatry Res. (1989) 28(2):193–213. doi: 10.1016/0165-1781(89)90047-4.

166. Suleiman KH, Yates BC, Berger AM, Pozehl B, Meza J. Translating the Pittsburgh Sleep Quality Index into Arabic. West J Nurs Res. (2010) 32(2):250–268. doi: 10.1177/0193945909348230.

167. Cohen S, Kamarck T, Mermelstein R. A global measure of perceived stress. J Health Soc Behav. (1983) 24(4):385–396. doi: 10.2307/2136404.

168. Norton R. Measuring marital quality: a critical look at the dependent variable. J Marriage Fam. (1983) 45:141–151. doi: 10.2307/351302.

169. Derogatis LR. SCL-90-R: Symptom Checklist-90-R: Administration, Scoring, and Procedures Manual. Minneapolis, MN: NCS Pearson. (1975).

170. Oranje AP, Glazenburg EJ, Wolkerstorfer A, De Waard-van der Spek FB. Practical issues on interpretation of scoring atopic dermatitis: the SCORAD index, objective SCORAD and the three-item severity score. Br J Dermatol. (2007) 157(4):645–648. doi: 10.1111/j.1365-2133.2007.08112.x.

171. Goodman R. The Strengths and Difficulties Questionnaire: a research note. J Child Psychol Psychiatry. (1997) 38(5):581–586. doi: 10.1111/j.1469-7610.1997.tb01545.x.

172. Aydemir Ö. Konsültasyon-Liyezon psikiyatrisinde yaşam kalitesi ölçümü: Kısa Form-36 (SF-36). Psikiyatri Psikoloji Psikofarmakol Derg. (1999) 7(2):14–23.

173. Hays RD, Morales LS. The RAND-36 measure of health-related quality of life. Ann Med. (2001) 33(5):350–357. doi: 10.3109/07853890109002089.

174. Bullinger M, Kirchberger I. SF-36-Fragebogen zum Gesundheitszustand (PSYNDEX Tests Review). MOS Short-Form-36 Health Survey (SF-36). Göttingen: Hogrefe. (1998).

175. Ware JE. SF-36 Health Survey: manual and interpretation guide. Boston, MA: The Health Institute. (1993).

176. Barnett BE, Hanna B, Parker G. Life event scales for obstetric groups. J Psychosom Res. (1983) 27(4):313–320. doi: 10.1016/0022-3999(83)90054-5.

177. Ainsworth MDS, Blehar MC, Waters E, Wall S. Patterns of attachment: a psychological study of the strange situation. Hillsdale, NJ: Erlbaum. (1978). doi: 10.4324/9780203758045.
